# Glycan characteristics of human heart constituent cells maintaining organ function: relatively stable glycan profiles in cellular senescence

**DOI:** 10.1007/s10522-021-09940-z

**Published:** 2021-10-12

**Authors:** Yoko Itakura, Norihiko Sasaki, Masashi Toyoda

**Affiliations:** Tokyo Metropolitan Institute of Gerontology, 35-2 Sakae-cho, Itabashi-ku, Tokyo, 173-0015 Japan

**Keywords:** Glycan profile, Lectin microarray, Cell surface protein, Cardiomyocyte, Cardiac fibroblast, Vascular endothelial cell

## Abstract

Cell surface glycoproteins, which are good indicators of cellular types and biological function; are suited for cell evaluation. Tissue remodeling using various cells is a key feature of regenerative therapy. For artificial heart remodeling, a mixture of heart constituent cells has been investigated for organ assembly, however, the cellular characteristics remain unclear. In this study, the glycan profiles of human cardiomyocytes (HCMs), human cardiac fibroblasts (HCFs), and human vascular endothelial cells (ECs) were analyzed using evanescent-field lectin microarray analysis, a tool of glycan profiling, to clarify the required cellular characteristics. We found that ECs had more “α1-2fucose” and “core α1-6fucose” residues than other cells, and that “α2-6sialic acid” residue was more abundant in ECs and HCMs than in HCFs. HCFs showed higher abundance of “β-galactose” and “β-*N*-acetylgalactosamine” residues on *N*-glycan and *O*-glycan, respectively, compared to other cells. Interestingly, cardiac glycan profiles were insignificantly changed with cellular senescence. The residues identified in this study may participate in organ maintenance by contributing to the preservation of glycan components. Therefore, future studies should investigate the roles of glycans in optimal tissue remodeling since identifying cellular characteristics is important for the development of regenerative therapies.

**Supplementary Information:**

The online version contains supplementary material available at 10.1007/s10522-021-09940-z.

## Introduction

Cellular conditions change based on the surrounding environment. Glycans on cell surface proteins are good indicators of cellular types and biological function. For example, cell surface glycoproteins differ based on cell types (Tao et al. 2008; Pilobello et al. 2007; Tateno et al. 2010; He et al. 2010) and change with differentiation; for instance embryonic stem cells to embryoid bodies and induced-pluripotent stem cells to cardiomyocyte (Alvarez-Manilla et al. 2010; Amano et al. 2010; Konze et al. 2017); these changes are associated with functional properties (Tateno et al. 2016; Toyoda et al. 2011; Kuno et al. 2008). We have previously reported that the abundance of sialic acids on membrane glycoproteins decreased with cellular senescence in human skin dermal fibroblasts, and that the reduction of sialic acids inhibited differentiation into myofibroblast (Itakura et al. 2016; Sasaki et al. 2017); therefore, glycan alterations with cellular senescence influence cellular functions. Moreover, the altered ratio of sialic acids on the membrane to the intracellular glycoproteins is indicative of not only cellular senescence but also of aging (Itakura et al. 2018). The influence of glycans can also be illustrated using various glycan analysis. Glycomics and glycoproteomics, using tissues and cells, are used for immunological recognition and cancer diagnosis (Mahal 2008; Narimatsu et al. 2018). Although glycans influence biological functions, the specific glycans in each cell type and the mechanism underlying glycan changes during cellular senescence or human aging remain unknown. Moreover, glycan changes are also implicated in the biological functions of peripheral cells.

Such cell characteristics influence regenerative therapy. Studies on regenerative therapies, using cell-based techniques, cell-free biomaterial, and cell-scaffold hybrid approaches, are anticipated in disease treatment (Tomov et al. 2019); identifying the characteristics of cells used in such therapies is essential. Tissue engineering using heart tissue cells is one of the main approaches in regenerative therapy. The heart tissue differs of the other organs since its ability to maintain function through its lifespan is higher than that of other tissues without the need for regeneration. The characteristic cells with high ability to maintain function with cellular senescence remains unclear. However, senescence of various cells that constitute cardiac vessels may induce cardiovascular disease in vivo (Childs et al. 2018). A senescent study of mouse cardiomyocyte in vitro has been investigated as a model system for translational research of human cardiology (Wang et al. 2016; Haseli et al. 2020; Hoes et al. 2019). The senescence of mouse cardiomyocytes occurs with extended cultivation, as shown by increasing levels of senescence-associated β-galactosidase (SA-β-galactosidase) and cell cycle regulators. Thus, it is important to evaluate cells used for cardiac engineering to determine if the accumulation of aged cells affects the overall aging process of an individual. Recently, to assemble tissues with high efficacy, a mixture of multiple cell types is used to construct 3D structures (Maiullari et al. 2018). Cardiomyocytes (CMs), cardiac fibroblasts (CFs), and vascular endothelial cells (ECs) are often used for cardiac modeling, although their viability and functionality differ based on their respective proportions in cellular mixture (Noguchi et al. 2016; Moldovan 2018). Therefore, it is necessary to understand the characteristics of the heart constituent cells.

In this study, to identify the glycan conditions of cells used for tissue assembly, we investigated the glycan alterations with cellular senescence in human cardiomyocytes (HCMs), human cardiac fibroblasts (HCFs), human coronary artery endothelial cells (HCAECs), and human microvascular endothelial cells (HMVECs), as well as the glycan profiles of their cellular proteins. Glycan profiles were analyzed using evanescent-field lectin microarrays, a suitable tool used for glycan profiling. Our study provides insights into the cell characteristics that influence maintenance of function and further contributes to the standardization of cells used for transplantation and tissue assembly.

## Materials and methods

### Cell culture

Fetal human cardiomyocytes (HCMs) were purchased from ScienCell Research Laboratories (Carlsbad, CA, USA). Human cardiac fibroblasts (HCFs) derived from a 15-year-old subject were purchased from Cell Systems (Kirkland, WA, USA). Human coronary artery endothelial cells (HCAECs) derived from a 48-year-old subject and human microvessel endothelial cells (HMVECs) derived from the heart of a 35-year-old subject were purchased from the Lonza (Walkersville, MD, USA). The population doubling levels (PDLs) of the purchased HCMs, HCFs, HCAECs, and HMVECs at the first seeding were defined as “0”. Cell proliferative capacity was assessed by calculating total PDL using the following formula: PDL = log_2_(total number of cells/initial number of cells). PDL counts were rounded up to the nearest whole number. Each purchased cell line was cultured, and stocks of frozen cells were prepared for the experiments. The PDL of each cell line at the time of freezing was set as the initial value. HCMs were cultured in Cardiac Myocyte Medium (CMM) (ScienCell, #6201) supplemented with Cardiac Myocyte Growth Supplement (CMGS), 25 mL fetal bovine serum (FBS) (ScienCell Research Lab.), and 50 U/mL penicillin along with 50 µg/mL streptomycin (Gibco, Grand Island, NY, USA). HCFs were cultured in Cell Systems Complete Medium with Attachment Factor containing FBS, Defined Cell Boost (Cell Systems, #CS4Z0500R), and 50 U/mL penicillin and 50 µg/mL streptomycin (Gibco). HCAECs and HMVECs were cultured in EGM-2MV with supplements and growth factors (#CC-3202, Lonza). In general, cell culture was performed following the manufacturer’s instructions. Just before reaching confluence for proliferation, the cells were sub-cultured using approximately 1 × 10^5^ to 4 × 10^5^ cells in HCMs and HCFs, and approximately 1 × 10^4^ cells in HCAECs and HMVECs in 60 mm or 100 mm plastic dishes (BD Falcon, San Jose, CA, USA) at 37 °C under a humidified atmosphere with 5% CO_2_. All cultures were performed in biological triplicate. Cell pellets were collected to analyze the specificities of the cells (PDLs: HCMs, 16–45; HCFs, 16–31; HCAECs, 13–37; HMVECs 12–19).

### Senescence-associated β-galactosidase (SA-β-galactosidase) detection

SA-β-galactosidase activity in cultured cells was detected histochemically using the Senescence Detection Kit (Calbiochem, EMD Biosciences, Darmstadt, Germany). In brief, culture medium was removed, the cultured cells were washed with phosphate-buffered saline (PBS), and then fixed with Fixative Solution at 25 °C for 15 min. After rinsing with PBS, the cells were stained overnight with the staining solution (Staining solution: Staining supplement: 20 mg/mL X-gal, 94:1:5) at 37 °C. After incubation, the stained cells were observed under a microscope.

### Protein extraction

Cell cultures of all cell lines were harvested during growth and after growth arrest. Total proteins were extracted from HCMs, HCFs, HCAECs, and HMVECs (approximately 4 × 10^4^ to 1 × 10^6^ cells) collected at each proliferative phase using the CelLytic MEM Protein Extraction kit (Sigma-Aldrich, St. Louis, MO, USA) as described previously (Itakura et al. 2011, 2013). Briefly, 300 µL cold Lysis Buffer containing 1% protease inhibitor cocktail was added to the cells and the supernatant was collected. After incubation at 30 °C for 5 min, the supernatant was separated into the upper hydrophilic and the lower hydrophobic phases and centrifuged at 3000×*g* at 25 °C for 5 min; the upper and lower phase solutions were then collected separately. Each protein concentration was determined using the Micro BCA Protein Assay kit (Thermo Fisher Scientific Inc., Waltham, MA, USA).

### Lectin microarray analysis

Lectin microarray analyses of the hydrophobic and hydrophilic protein extracts were performed as described previously (Kuno et al. 2008; Itakura et al. 2011, 2018). Briefly, 0.2 µg of total proteins, including glycoproteins, were labeled with Cy3 mono-reactive dye (GE Healthcare, Buckinghamshire, UK) through incubation in PBS at 25 °C for 1 h. The excess dye was removed using a spin-type column loaded with Sephadex G-25 fine matrix (GE Healthcare) and the collected Cy3-labeled glycoprotein solution was diluted to 2 µg/mL with probing buffer (Tris-buffered saline containing 1% Triton X-100, 1 mM CaCl_2,_ and 1 mM MnCl_2_, pH 7.4). The glycoprotein solution (0.5 µg/mL) was then applied to a LecChip (ver.1.0; GlycoTechnica, Yokohama, Japan) (Supplemental Table S1). After incubation at 4 °C for approximately 17 h, the reaction solution was discarded. The LecChip was washed three times with probing buffer before scanning using the evanescent-field fluorescence scanner, GlycoStation^TM^ Reader 1200 (GlycoTechnica). Each sample was measured in triplicate. Data were analyzed using GlycoStation^TM^ Tools Signal Capture 1.0 and GlycoStation^TM^ Tools Pro 1.0 (GlycoTechnica). For accurate analysis, the data were used with average-normalization (Tateno et al. 2010). The details of lectin microarray are shown in Supplemental Table S1 and the original data of membrane and intracellular glycoproteins after normalizing for chip are presented in Supplemental Tables S2 and S3, respectively.

### Lectin blot analysis of whole extracts and membrane glycoproteins from heart constituent cells

One microgram of the whole protein extracts from HCMs (PDL 16), HCFs (PDL 16), HCAECs (PDL 13), and HMVECs (PDL 12), and 0.5 µg of hydrophobic protein extracts from HCMs (PDLs 16 and 45), HCFs (PDLs 16 and 31), and HCAECs (PDLs 13 and 37) were separated on 12.5% SDS-PAGE gel. The proteins were then transferred to a PVDF membrane and incubated overnight with PVDF Blocking Reagent for Can Get Signal (TOYOBO Co. Ltd., Osaka, Japan) at 4 °C. After blocking, the membrane was washed with Tris-buffered saline containing 1% Tween 20 (TBST) and reacted with each of 1 µg/mL biotinylated SNA (EY Laboratories Inc., San Mateo, CA, USA), 2 µg/mL biotinylated WFA (EY Laboratories Inc.), 1 µg/mL biotinylated RCA120 (Vector Laboratories Inc., Burlingame, CA, USA), 1 µg/mL biotinylated AAL (J-OIL MILLS Inc., Tokyo, Japan), 2 µg/mL biotinylated TJA-II (Seikagaku Biobusiness, Tokyo, Japan), 1 µg/mL biotinylated AOL (Tokyokasei Industry Co., LTD., Tokyo, Japan), and 1 µg/mL biotinylated PHA-E (EY Laboratories Inc.) at 25 °C for 1 h. Before use, TJA-II and AOL were biotinylated using a Biotin Labeling Kit-NH_2_ (DOJINDO, Kumamoto, Japan). The membrane was then incubated with horseradish peroxidase-conjugated streptavidin (1:5000; Jackson Immuno Research Laboratories Inc., West Grove, PA, USA) at 25 °C for 30 min, followed by reaction with ECL^TM^ Prime Western Blotting Detection Reagents (Global Life Sciences Technologies Japan, Tokyo, Japan) for 3 min. The proteins were visualized and measured on the Fusion SOLO.7 S.EDGE system (M&S Instruments Inc., Osaka, Japan).

### Immunocytochemistry for each cell type

HCMs, HCFs, and HCAECs (PDLs 28, 24, and 11, respectively) were fixed with 4% paraformaldehyde and washed with PBS. To analyze the cellular glycan characteristics, the fixed cells were incubated with BlockAid Blocking Solution (Thermo Fisher Scientific Inc.) at 25 °C for 20 min, followed by reaction with anti-S100A4 antibody for FSP1 (Abcam plc, Cambridge, UK, #ab27957, 1:200), anti-CD31 antibody (Proteintech Group Inc., Rosemont, IL, USA, #11265-1-AP, 1:50), and a mixture of anti-cTnI antibody (HyTest Ltd., Turku, Finland, #4T21, 1:20) and 10 µg/mL biotinylated SNA (Vector Laboratories Inc., Burlingame, CA, USA) in PBS for 30 min in the dark. After washing, cells were reacted with a mixture containing anti-Rabbit IgG-AF546 (Thermo Fisher Scientific Inc., 1:500) and fluorescein isothiocyanate conjugated WFA (Vector Laboratories Inc., 1:200) or UEA-I (Vector Laboratories Inc., 1:200) in PBS, in combination with S1004A or CD31, respectively, and with anti-mouse IgG-AF488 (Thermo Fisher Scientific Inc., 1:500) and Texas Red conjugated streptavidin (Vector Laboratories Inc., 1:100) in PBS, in combination with cTnI at 25 °C for 20 min. For single staining with lectins, blocked cells with blocking solution were reacted with biotinylated AAL (1:100), AOL (1:100), and TJA-II (1:100) in PBS at 25 °C for 30 min in the dark. After washing, cells were reacted with AF647 conjugated streptavidin (Thermo Fisher Scientific Inc., 1:100) in PBS in combination with each biotinylated lectin, or fluorescein isothiocyanate conjugated RCA120 (Vector Laboratories Inc., 1:200) at 25 °C for 20 min. After rinsing, the cells were stained with DAPI (FUJIFILM Wako Pure Chemical Corporation, Osaka, Japan, 1:500) in PBS for 10 min, followed by three 5 min rinses with PBS. The cells were then mounted using Mounting Medium (Dako, Agilent Technologies, Inc., Santa Clara, CA, USA). The stained cells were observed under a microscope (TCS SP8 DLS, Leica Microsystems, Tokyo, Japan) and compared. HCMs were fixed at T1 and T2 (PDLs 28 and P45, respectively). To identify the cellular characteristics, the fixed cells were blocked with BlockAid Blocking Solution containing 0.2% Triton X-100 and then reacted with anti-cTnI antibody at 25 °C for 30 min in the dark in PBS containing 0.1% Triton X-100 (PBSTx). The cells were reacted with anti-mouse IgG AF488 in PBSTx at 25 °C for 20 min. After washing, the cells were stained with DAPI in PBSTx as mentioned above.

### Gene expression

Total RNA was isolated from each cell using RNeasy plus mini kit (QIAGEN, Hilden, Germany) and subsequently reverse-transcribed using ReverTra Ace qPCR RT Master Mix (Toyobo Co. Ltd., Osaka, Japan). Real-time PCR was performed using the Power SYBR^TM^ Green PCR Master Mix (Applied Biosystems, Forster City, CA, USA) and StepOnePlus^TM^ real-time PCR system (Applied Biosystems). *GAPDH* was amplified and used as the internal control. The threshold crossing value was noted for each transcript and normalized to that of the internal control. Relative quantitation of each mRNA was performed using the comparative Ct method. The primer sets used for real-time PCR were as follows: *GAPDH*, 5′-GCTCAGACACCATGGGGAAGGT-3′ (forward) and 5′-GTGGTGCAGGAGGCATTGCTGA-3′, (reverse); *cTnI*, 5′-CCGTGTGGACAAGGTGGATG-3′ (forward) and 5′-TTAAACTTGCCTCGAAGGTCAAAGA-3′ (reverse); and *GATA4*, 5′-TCCAAACCAGAAAACGGAAGC-3′ (forward) and 5′-GCCCGTAGTGAGATGACAGG-3′ (reverse).

### Statistical analysis

Significant differences between young and senescent cells were evaluated using the Student’s *t*-test, performed on IBM SPSS Statics26 (Stats Guild Inc., Tokyo, Japan). A *p* < 0.05 was considered statistically significant. All experiments were performed with repetition.

## Results

### Cellular characteristics of the heart constituent cells

**Fig. 1:**
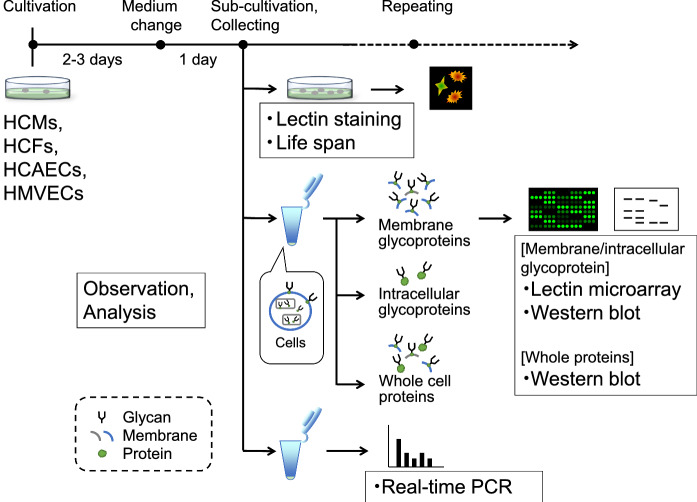
Schematic illustration of cellular characteristics analysis

**Fig. 2:**
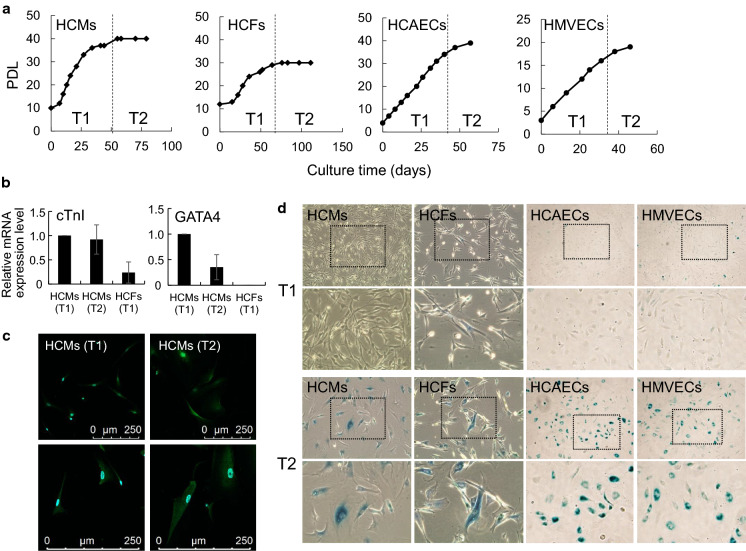
Cellular characteristics of human heart constituent cells (HCMs, HCFs, HCAECs, and HMVECs). **a** The representative proliferations of each cell line (HCMs, HCFs, HCAECs, and HMVECs) are shown as PDLs. Two phases were separated into growth phase (T1) and growth arrest (T2) for each cell line. **b** Comparison of HCMs with HCFs in gene expression of cardiomyocyte markers (cTnI and GATA4). Bar graph representing the relative expression level with reference to GAPDH at T1 and T2. The data are presented as the mean ± SD (n = 3). **c** Protein expression of the characteristic marker (cTnI) in cardiomyocytes. HCMs at T1 (left) and T2 (right) are shown with a cardiomyocyte marker (cTnI, green), and nucleus (DAPI, blue) as the overlay image. The bottom panels show a magnified image. Scale bar = 250 μm. **d** HCMs, HCFs, HCAECs, and HMVECs at T1 and those at T2 are shown with SA-β-galactosidase. The bottom panels show a magnified image of the squared area in the upper panels (× 10). All cultures were performed in biological triplicate

To obtain the aged cells of the four cell types, HCMs, HCFs, HCAECs, and HMVECs, we cultivated cells until growth arrest and analyzed cells at several time points (Fig. 1). The representative growth curves for these cell types are shown in Fig. 2a. Growth arrests of HCMs, HCFs, HCAECs, and HMVECs were observed at approximately PDLs 40, 30, 40, and 20, respectively (Fig. 2a). To determine cellular characteristics, we divided the growth phases of each cell type into the growth phase (T1) and growth arrest (T2) (Fig. 2a). CMs are typically cultivated under the confluence condition in vitro, however, the HCMs used in this study were passaged before reaching confluence, appropriate for long sub-cultivation. The characteristics of senescent HCMs were evaluated based on the gene expression of the cardiomyocyte marker *cTnI* and the transcription factor *GATA4* and confirmed using immunostaining for cTnI (Fig. 2b and c). The mRNA and protein of cTnI in HCMs were expressed through aging. On the contrary, the mRNA expression of GATA4 decreased at T2. Both mRNA expressions in HCMs differed from those in HCFs. HCMs, HCFs, HCAECs, and HMVECs at T1 were slightly stained with SA-β-galactosidase, whereas those at T2 were noticeably stained (Fig. 2d). These results indicate that each selected cell types can be sub-cultured under certain conditions in vitro and that these cells age when reaching the growth arrest.

### Glycan profiles of HCMSs, HCFs, HCAECs, and HMVECs

**Fig. 3:**
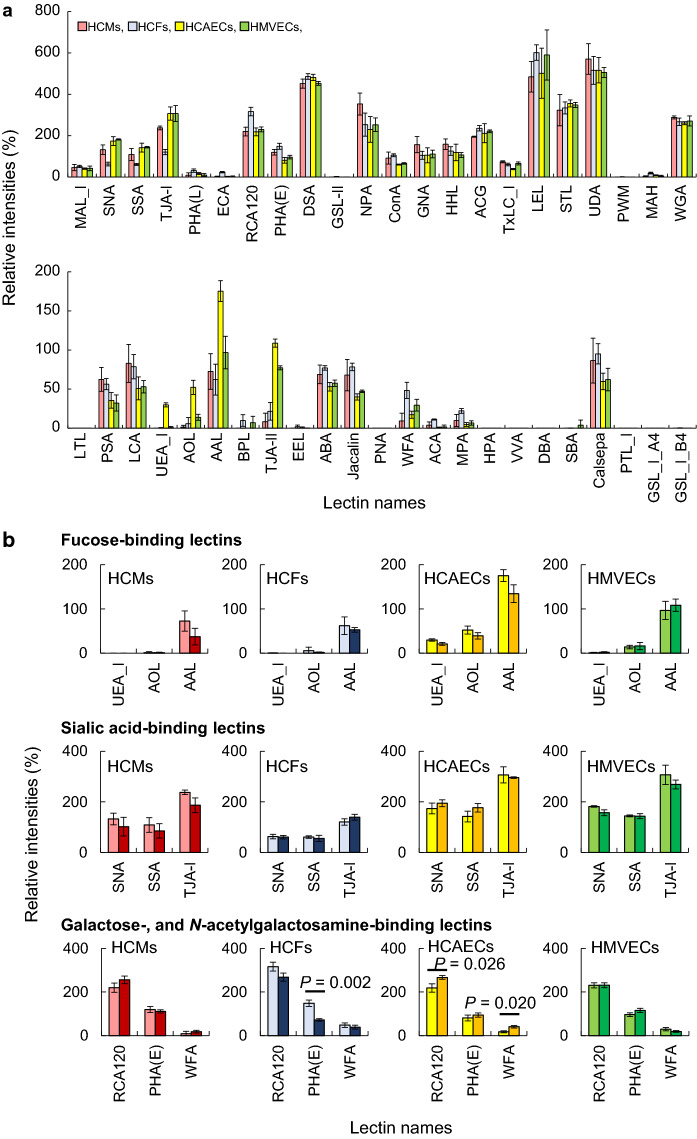
Membrane glycan profiles in HCMs, HCFs, HCAECs, and HMVECs, analyzed using lectin microarray. **a** Bar graphs representing the signal intensities (%) for 45 lectins in HCMs (red), HCFs (blue), HCAECs (yellow), and HMVECs (green) at T1 (growth phase). The data are presented as the mean ± SD (n = 3). **b** Bar graphs representing the signal intensities (%) in HCMs (red), HCFs (blue), HCAECs (yellow), and HMVECs (green) at T1 (growth phase; light color) and T2 (growth arrest; dark color). The selected signal intensities of four binding-type (fucose, sialic acid, galactose, and *N*-acetylgalactosamine) lectins were observed (P < 0.05 between T1 and T2 based on Student’s t-tests). The glycan profiles of 45 lectins are shown in Supplemental Fig. S1. The data are presented as the mean ± SD (n = 3). The characteristics of the 45 lectins and the values of the signal intensities obtained in this analysis are shown in Supplemental Tables S1 and S2, respectively. The data of T1 in b are same as in a. All experiments were performed in triplicate

To evaluate the glycan profiles associated with the points T1 and T2, lectin microarray analysis was performed for HCMSs, HCFs, HCAECs, and HMVECs (Supplemental Table S2). In T1, each cell type showed different glycan profiles on their respective membrane glycoproteins (Fig. 3a). Human ECs are recognized by UEA-I. The signal intensities of UEA-I (α1-2fucose-binding lectin) in HCAECs and TJA-II (α1-2fucose-binding lectin) in both HCAECs and HMVECs were significantly higher than those in other cell types. The signal intensities of PSA and LCA (α1-6fucose- and α-mannose-binding lectins) in both HCMs and HCFs were higher than those in HCAECs and HMVECs, whereas the signal intensities of AOL and AAL (core α1-6fucose-binding lectins) in HCAECs were higher than those in the other cell types. The signal intensities of mannose-binding lectins, such as NPA, GNA, and HHL in HCMs were slightly higher than those in the other cell types but those with similar type lectins, such as ConA and Jacalin, were high in HCMs and HCFs. The signal intensities of SNA, SSA, and TJA-I (α2-6sialic acid-binding lectins) in HCMs, HCAECs, and HMVECs were higher than those in HCFs. On the contrary, the signal intensities of RCA120, ECA, PHA-L, and PHA-E (galactoseβ1-4-*N*-acetylgalactosamine-binding lectins), which do not permit the sialic acid residues or prefer exposed galactose residues, in HCFs were the highest among the four cell types. Moreover, the signal intensities of WFA (LacdiNAc, *N*-acetylgalactosamineβ1-4-*N*-acetylglucosamine, structure-binding lectin), ACA, and MPA (galactoseβ1-3-*N*-acetylgalactosamine-binding lectins) in HCFs were also significantly higher than those in the other cell types. The signal intensities of DSA, LEL, STL, UDA, and WGA (chitin, *N*-acetylglucosamine oligomer, -binding lectins) were high in all cell types. These results suggested that the four cell types in heart constituent cells have various *N*- and *O*-glycans on their cell surface. As a characteristic feature, HCMs had higher abundance of sialic acid and mannose residues, while HCFs had more β-galactose residues and the LacdiNAc structure. Moreover, the results also suggested that HCAEC and HMVEC had higher abundances of absolute α1-2fucose and core α1-6fucose, and sialic acid residues. Thus, these results suggested the existence of specific glycan features in each cell type constituting cardiac tissues, despite the four glycan profiles being similar.

To investigate the glycan changes in senescent cells of each cell type, the glycan profiles in HCMs, HCFs, HCAECs, and HMVECs were compared (Supplemental Fig. S1). In the case of the growth arrested state (T2), HCMs showed decreased signal intensities of AAL, SNA, SSA, and TJA-I (Fig. 3b). However, no significant differences were observed between the signals of their lectins at T1 and T2. HCFs showed little change in the signal intensities of fucose- and sialic acid-binding lectins. Conversely, HCFs showed a decrease in the signal intensities of RCA120, though not significant, and PHA-E, suggesting the existence of bisecting *N*-acetylglucosamine on *N*-glycan with β1-4galactose residues, unlike the other cells. HCAECs showed a decreased signal intensity of AAL, though not significant, and an increased signal intensity of RCA120. However, whether HCAECs showed slightly increased signal intensities of SNA and SSA, and decreased that of TJA-I, remained undetermined. The signal intensities of the lectins in HMVECs at T1 were as same as those at T2, suggesting that the specific glycans characterized by some lectins in each cell type showed only modest changes, without significant differences, in cellular senescence.

**Fig. 4:**
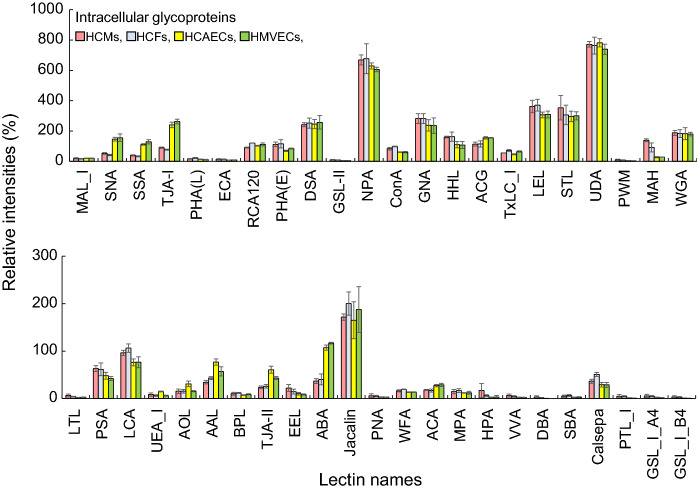
Intracellular glycan profiles in HCMs, HCFs, HCAECs, and HMVECs, analyzed using lectin microarray. Bar graph representing the signal intensities (%) of 45 lectins in HCMs (red), HCFs (blue), HCAECs (yellow), and HMVECs (green) at T1 (growth phase). The data are presented as the mean ± SD (n = 3). The characteristics of the 45 lectins and the values of the signal intensities obtained in this analysis are shown in Supplemental Tables S1 and S3, respectively. All experiments were performed in triplicate

Next, the glycan profiles of intracellular glycoproteins were analyzed using lectin microarray for aging (Supplemental Table S3), as previously reported (Itakura et al. 2018). The glycan profiles of each cell type for T1 are shown in Fig. 4. The signal intensities of PSA and LCA in HCMs and HCFs were slightly higher than those in HCAECs and HMVECs. The signal intensities of NPA, ConA, GNA, and HHL in HCMs and HCFs were also higher than those in both the ECs. On the contrary, the signal intensities of SNA, SSA, TJA-I, ACG (α2-3sialic acid-binding lectin) and ABA (galactoseβ1-3-*N*-acetylgalactosamine-binding lectin) in both HCAECs and HMVECs were significantly higher than those in HCMs and HCFs. Although the signal intensities of UEA-I in HCAECs and WFA in HCFs were quite unique in the membrane glycan profiles, the same was not observed in the intracellular glycan profiles. Moreover, the signal intensity of MAH (α2-3sialic acid on *O*-glycan-binding lectin), which was the highest in HCMs, followed by that in HCFs, was significantly different compared with those of the membrane glycoproteins. These results showed that intracellular glycans of HCMs and HCAECs were more similar to those of HCFs and HMVECs, respectively.

As for the membrane glycoproteins, we investigated the intracellular glycan profiles of HCMs, HCFs, HCAECs, and HMVECs at T2 and compared them with those at T1 (Supplemental Fig. S2). The signal intensities of MAH in HCMs and HCFs were decreased at T2. Although there were some changes in the signal intensities in the four cell types, there were no commonalities among the lectins in each cell type. These results suggested no significant changes in the glycosylation of intracellular glycoproteins as well as those of the respective membrane glycoproteins, except for α2-3sialic acid on *O*-glycans.

### Quantitative comparison of glycoprotein expression among cardiac constituent cells

**Fig. 5:**
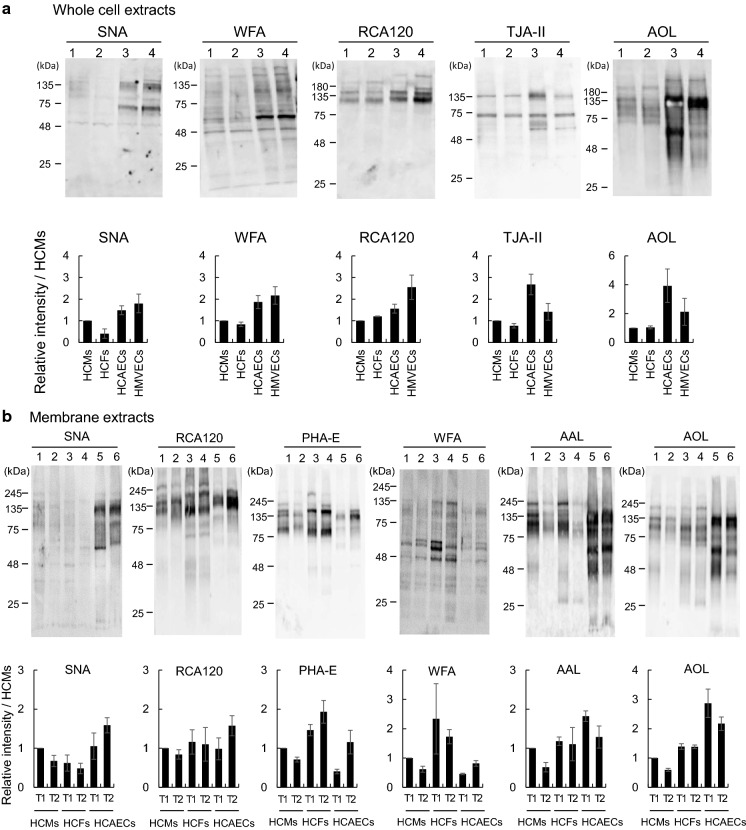
Lectin blot detection of whole cell and membrane extracts from heart constituent cells. **a** Whole cell extracts from HCMs, HCFs, HCAECs, and HMVECs were applied to lanes 1–4, respectively and were subjected to lectin blot analysis using biotinylated-SNA, -WFA, -RCA120, -TJA-II, and -AOL. **b** The membrane extracts from HCMs, HCFs, and HCAECs at T1 (growth phase) and the corresponding extracts at T2 (growth arrest) were applied to lanes 1, 3, 5 and 2, 4, 6, respectively and were subjected to lectin blot analysis using biotinylated-SNA, -RCA120, -PHA-E, -WFA, -AAL, and -AOL. Bar graph representing the bands for each lane after normalization to HCMs using the Fusion system. All experiments were performed with repetition. The data are presented as the mean ± SD (n = 3)

To compare the specific glycans expressed in heart constituent cells (HCMs, HCFs, HCAECs, and HMVECs), proteins extracted from each whole cell were applied to a lectin blot, to show the characteristics of the lectin microarray data (Fig. 5a). The extractions were also evaluated using silver staining (Supplemental Fig. S3a). Upon comparison of the four cell types at T1, the amount of proteins in HCFs that reacted with SNA was the least, followed by that in HCMs. In the case of WFA and RCA120, the intensities of the bands in HCMs and HCFs were approximately equal, and those in HCAECs and HMVECs were more intense than those in HCMs and HCFs. The most intense bands with TJA-II were observed in HCAECs. Similar to the blot for TJA-II, the intensities of the AOL bands in HCAECs, followed by those in HMVECs, were significantly greater than those in HCMs and HCFs. Glycoproteins with some lectins such as WFA and AOL were detected as broad bands in HCAECs and HMVECs, whereas other types of glycan on proteins detected with RCA120 were mostly detected at high molecular weights in the four cell types. These results suggested that the expression of glycans in whole cell extracts differed among the four cell types. Moreover, their expression levels were not similar to two individual (membrane and intracellular) glycan profiles.

To compare the specific glycan changes in heart constituent cells, each membrane glycoprotein was applied to a lectin blot with each cell type HCMs, HCFs, and HCAECs, except for HMVECs, whose glycan profile was barely changed between T1 and T2 (Fig. 5b). Representative lectins that were changed between T1 and T2, as shown by lectin microarray analysis during aging for specific cellular characteristics, were used for this analysis. The extractions were also evaluated by silver staining (Supplemental Fig. S3b). The intensities of the bands with SNA in HCMs and HCFs at T2 were slightly decreased compared to those at T1, but those in HCAECs were increased at T2. The intensities of the bands with RCA120 in HCFs were slightly decreased at T2 compared to those at T1, whereas those in HCAECs were increased at T2 compared to those at T1. The intensities of the bands with PHA-E in HCFs and HCAECs at T2 were increased compared to those at T1, whereas those in HCMs were slightly decreased at T2 compared to those at T1. The intensities of the bands with WFA in HCAECs at T2 were increased compared to those at T1. The intensities of the bands with AAL in HCMs and HCAECs at T2 were significantly decreased and those in HCFs were also slightly decreased. HCAECs with AAL showed a broad range of molecular weights for membrane glycoproteins, whereas HCMs and HCFs mainly displayed high-molecular weight glycoproteins, with different band patterns. The bands with low-molecular weights of 40–50 kDa in HCMs and HCFs disappeared with cellular senescence, whereas the bands at roughly 30 kDa in HCFs were retained. The detected AOL bands in the three cell types were very similar to the AAL bands. These results were in agreement with the lectin microarray data and suggested that the bands were quantitatively changed between T1 and T2 in membrane glycoproteins.

### Expression of characteristic glycans in HCMs, HCFs, and HCAECs

**Fig. 6:**
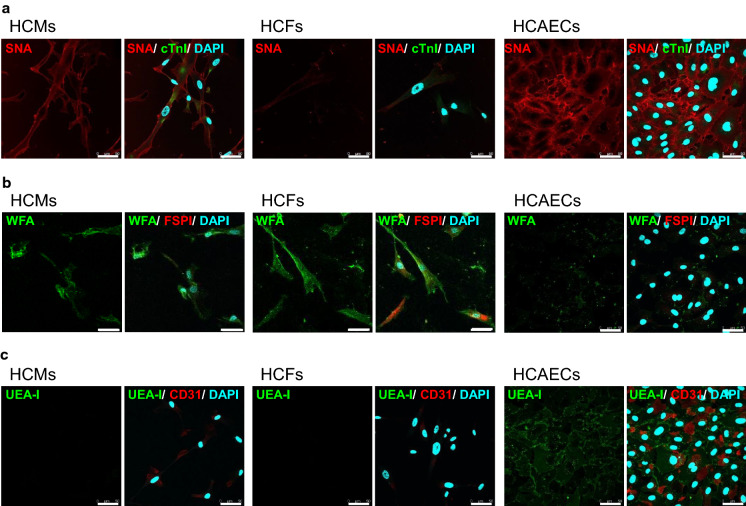
Localization of characterized glycans in heart constituent cells. HCMs (left), HCFs (middle), and HCAECs (right) at T1 stained with each of the characteristic lectins and cell characterizing markers. **a** Three cell types stained with SNA (red) and the overlay image (cTnI, green; right panel). **b** Three cell types stained with WFA (green) and the overlay image (FSP1, red; right panel). **c** Three cell types stained with UEA-I (green) and the overlay image (CD31, red; right panel). Blue staining represents the nucleus. Scale bar = 50 μm. All experiments were performed with repetition

To investigate the actual expression of some glycans on the cell surface as shown by lectin microarray analysis, three cell types at T1 were specifically stained for the lectins which had distinctive membrane glycan signals among the cell types, with cell-type specific markers as the controls. Highly stained cells with SNA were observed in HCMs and HCAECs (Fig. 6a). HCMs were confirmed by staining for cTnI, a myocardial marker (Supplemental Fig. S4a). On the contrary, slightly stained cells were observed in HCFs (Fig. 6a). When each cell type was stained with WFA, which was shown as a characteristic of HCFs by lectin microarray analysis (Fig. 3a), HCMs and HCAECs exhibited less staining, whereas HCFs were stained well (Fig. 6b). HCFs were confirmed by staining for FSP1, a fibroblastic marker (Supplemental Fig. S4b). Moreover, upon staining with UEA-I, HCAECs were the most strongly stained, whereas HCMs and HCFs were not stained (Fig. 6c). HCAECs were further confirmed by staining for CD31 (Supplemental Fig. S4c). To confirm the higher glycan expression, the three cell types were stained for other characteristic lectins (Supplemental Fig. S5). HCAECs were the most stained for by AAL staining, followed by HCMs and HCFs. Similar to the AAL staining, HCAECs had greater expression with AOL than the other cell types had. Upon staining with TJA-II, HCAECs were stained, whereas HCMs and HCFs were not stained. Moreover, highly stained cells with RCA120 were observed in HCFs, followed by HCMs and HCAECs. These results suggested that HCMs and HCAECs significantly expressed α2-6sialic acid, whereas HCFs barely expressed it, and that α1-2 and core α1-6fucose residues were specifically expressed in HCAECs as shown in the lectin microarray data. It was clearly observed that each cell type had characteristic features of glycans on its cell surface.

## Discussion

Cells have individual glycan profiles indicating cell types and directionality of differentiation (Pilobello et al. 2007; Toyoda et al. 2011; Itakura et al. 2013; Tateno et al. 2011; Kuno et al. 2008). Notably, the Glycome Atlas has been developed to allow visualization of local glycan profiling data from human and mouse (Konishi and Aoki-Kinoshita 2012; Nagai-Okatani et al. 2019a), and thus, cell clustering in tissues of these organism can be elucidated using glycans. In recent cardiac tissue engineering approaches, various methods have been used (Schwach and Passier 2019; Tomov et al. 2019; Maiullari et al. 2018; Moldovan 2018). For instance, an appropriate decellularization approach for organ generation was assessed using rat heart and kidney (Kawasaki et al. 2015); the combination of soft repair cells and porous scaffold materials prevents left ventricle dilatation (Geng et al. 2018). As a result of these methods, a supportive environment of cardiomyocyte was created in a combination with scaffold and cells, and vascularized by scaffold implantation (Radisic et al. 2008); cardiac performance was significantly improved following transplantation of allogeneic amniotic membrane-derived mesenchymal stromal cells into a porcine heart affected by chronic myocardial ischemia (Kimura et al. 2012). In clinical research, cellular sheet constructs have been used in patients with myocardial infarcts. Meanwhile, the implantation of cardiosphere-derived cells isolated from neonatal mice did not improve cardiac function (Zhao et al. 2018b). Thus, various approaches have been investigated, but the efficacy is different due to treatment complexity. Cell fusion approaches for artificial tissue remodeling have a remarkable potential owing to the resemblance with the cardiac tissue milieu and high availability. Noguchi et al. generated spheroids constructed from CMs, CFs, and ECs, and fused them with cardiac patches, which were grafted into a rat’s heart, and confirmed the formation of a vascular network within the transplanted grafts (Noguchi et al. 2016). Therefore, we analyzed the glycan features to evaluate the cardiac constituent cells used cardiac engineering.

The glycan profiles of HCAECs and HMVECs were similar to, but differed from HCMs and HCFs. The presence of α1-2fucose residues recognized by UEA-I was confirmed in HCAECs. ECs mainly expressed core α1-6fucose residues, whereas other types of fucosylation (such as α1-3fucose residues) were more distinctive in HCMs and HCFs. Core α1-6fucosylation of membrane glycoproteins in HCMs was hardly observed in the lectin microarray data, whereas AOL bands were detected in whole cell extracts from HCMs, suggesting the existence of fucose residues in intracellular or partially membranous glycoproteins. Cardiomyocytes derived from human induced pluripotent stem cells (iPSCs) have core α1-6fucose and α1-2fucosilated β1-3galactose residues on *N*-glycans on cell surface (Konze et al. 2017). In our study, the α1-6fucosylation and α1-2fucosylation are reflected in a signal of AAL and TJA-II (not UEA-I), respectively. Konze et al. suggested that bisecting *N*-acetylglucosamine structures emerge on immature CMs. Herein, we observed signals for PHA-E which suggests the existence of bisecting *N*-acetylglucosamine in HCMs. Considering the findings of Konze et al., contamination of immature CMs is a possibility, since the HCMs used in this study were derived from a fetus.

WFA binding is upregulated in cardiac hypertrophy model mice, as determined using staining assays (Nagai-Okatani et al. 2019b). An increase in WFA binding often suggests elevated expression of LacdiNAc structures, including β1-4*N*-acetylgalactosamine residues, or of carrier products from abnormal cells. Our data suggested that β1-4*N*-acetylgalactosamine residues increase with the self-replication of HCFs participating in fibrosis, considering the increase in relevant secretory proteins in the subject.

Both membrane and intracellular glycan profiles in cardiac constituent cells during aging were not significantly changed compared to those in other organs such as skin fibroblasts, which show decreased α2-6 and α2-3sialic acids residues with suppression of fibrosis (Itakura et al. 2016, 2018; Sasaki et al. 2017). In the case of HCMs, Kawamura et al. showed that α2-6sialylation was higher than α2-3sialylation on *N*-glycans obtained from human iPSC-derived CMs and HCMs, and that α2-6sialylation showed no change during differentiation (Kawamura et al. 2015). In our study, α2-6sialic acids residues were more abundant than α2-3sialic acids residues on membrane glycoproteins in HCMs. The slight reduction without a significant difference in sialic acids observed with cellular senescence in HCMs, as well as altered skin fibroblast characteristics, may indicate their function as suppressors of fibrosis in the cellular level. However, we speculate that the change of sialic acid residues is too small to inhibit fibrosis of the organ as the heart.

These results indicate insignificant characteristics in each cardiac constituent cell. The cardiac tissue has a greater ability than other organs to maintain function throughout life without the need for regeneration as mentioned in the Introduction. These suggest that the long-term survival of cells without significant cellular changes was necessary for the heart. We think that the heart has a role to play in continuing to work throughout life, and the in constantly trying to make the cells work to maintain function, like remodeling. And it may be only when they fail that heart failure occurs. At the same time, according to our study, the evaluation of glycosylation patterns can be discriminated between ECs and other type cells in fucosylation and between HCFs and others in sialylation. During tissue engineering, multiple cell types can be mixed and fused to facilitate tissue remodeling. Identification of the cellular conditioning markers such as each characteristic glycans will accelerate the selection of cell types with efficient differentiation potential, engraftment rate, purity, and other valuable features. On the contrary, α1-6fucosylation of *N*-glycans on cardiomyocyte proteins is reported to increase in diabetic model mice (Zhao et al. 2018a). The presence of α1-6fucose residues is a characteristic feature of HCMs and these residues are relevant to various cardiac diseases. Glycan signatures have already been used in cell selection for the removal of undifferentiated human pluripotent stem cells after induction of differentiation and determination of cell fate (Tateno et al. 2015). Moreover, the evaluation system, using the characterization of glycans, is anticipated in cell-based therapies, such as supplementing the decreasing population of chondrocytes with aging to determine cellular characteristics (Demoor et al. 2014).

Taken together, the glycan profiling in this study revealed the characteristic features of each type of cardiac constituent cells. Each cardiac tissue comprises different cellular ratios (Zhou and Pu 2016). Considering the optimal cellular types and ratios for tissue remodeling in patients-specific conditions based on the characteristic glycan indicators for each cell type could help increase the efficacy of tissue remodeling along with more appropriate cellular selection of disease relevance. In-depth understanding of glycan alterations in various cell types could also help to develop regenerative therapy.

## Conclusions

We demonstrated the glycan characteristics of human cardiac constituent cells, such as HCMs, HCFs, HCAECs, and HMVECs, using a lectin microarray. The glycan profile of each cell type showed insignificant changes with cellular senescence. Nevertheless, there are limitations to the present study, such as the use of cells derived from few subjects. A better understanding of each cellular condition is needed for advancing research on regenerative therapy due to differences in the various factors, including diseases and ages. In addition, further analysis of glycan composition via methods such as mass spectrometry will enhance understanding when combined with analysis of glycan structures, including branching, coordination, and binding modes (Shu et al. 2021). Investigating optimal cellular ratios for remodeling and the carrier proteins of characteristic glycans will be important in making these glycans useful as indicators for clarifying the details of personalized cardiovascular conditions and cellular functions in patients. Our findings may help in the development of medical treatments and tissue engineering for cardiovascular diseases.

## Supplementary Information

Below is the link to the electronic supplementary material.

Supplementary material 1 (PDF 1122 kb)

Supplementary material 2 (PDF 88 kb)
