# Supplementary material for: Glycan characteristics of human heart constituent cells maintaining organ function: relatively stable glycan profiles in cellular senescence
Source: Biogerontology. 2021 Oct 12;22(6):623–37. doi: 10.1007/s10522-021-09940-z (PMC8566412; doi:10.1007/s10522-021-09940-z)
Supplement: Supplementary file 1 — Supplementary material 1 (PDF 1122 kb) [file 10522_2021_9940_MOESM1_ESM.pdf]

Supplemental Data

Journal name: *Biogerontology*

Title: Glycan characteristics of human heart constituent cells maintaining organ function: relatively stable glycan profiles in cellular senescence

Author names: Yoko Itakura, Norihiko Sasaki, and Masashi Toyoda*

*Correspondence: Masashi Toyoda,

Research Team for Geriatric Medicine (Vascular Medicine), Tokyo Metropolitan Institute of Gerontology, 35-2 Sakae-cho, Itabashi-ku, Tokyo 173-0015, Japan; E-mail: mtoyoda@tmig.or.jp; Tel.: +81-3-3964-3241

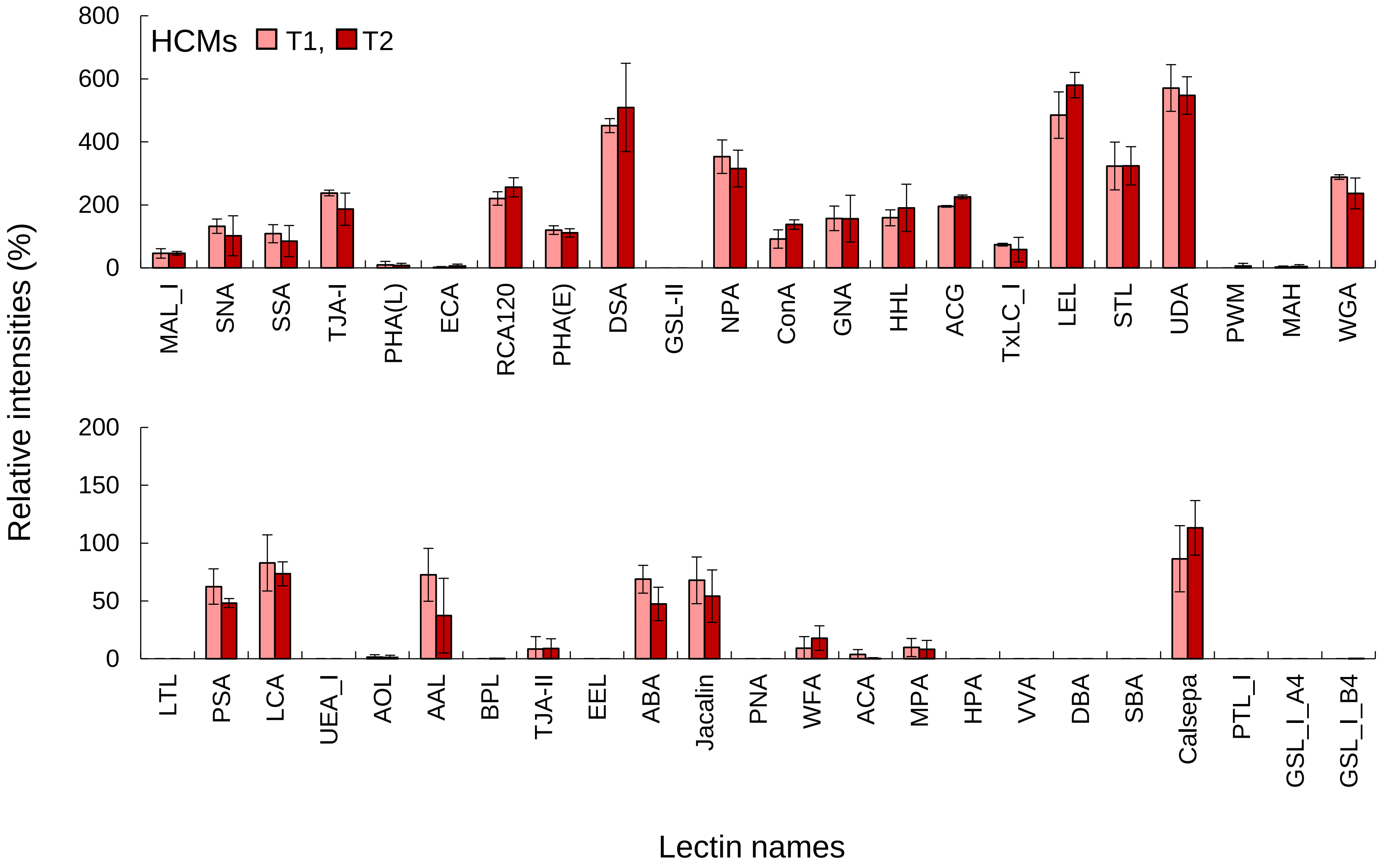

Supplemental Fig. S1 Itakura et al.

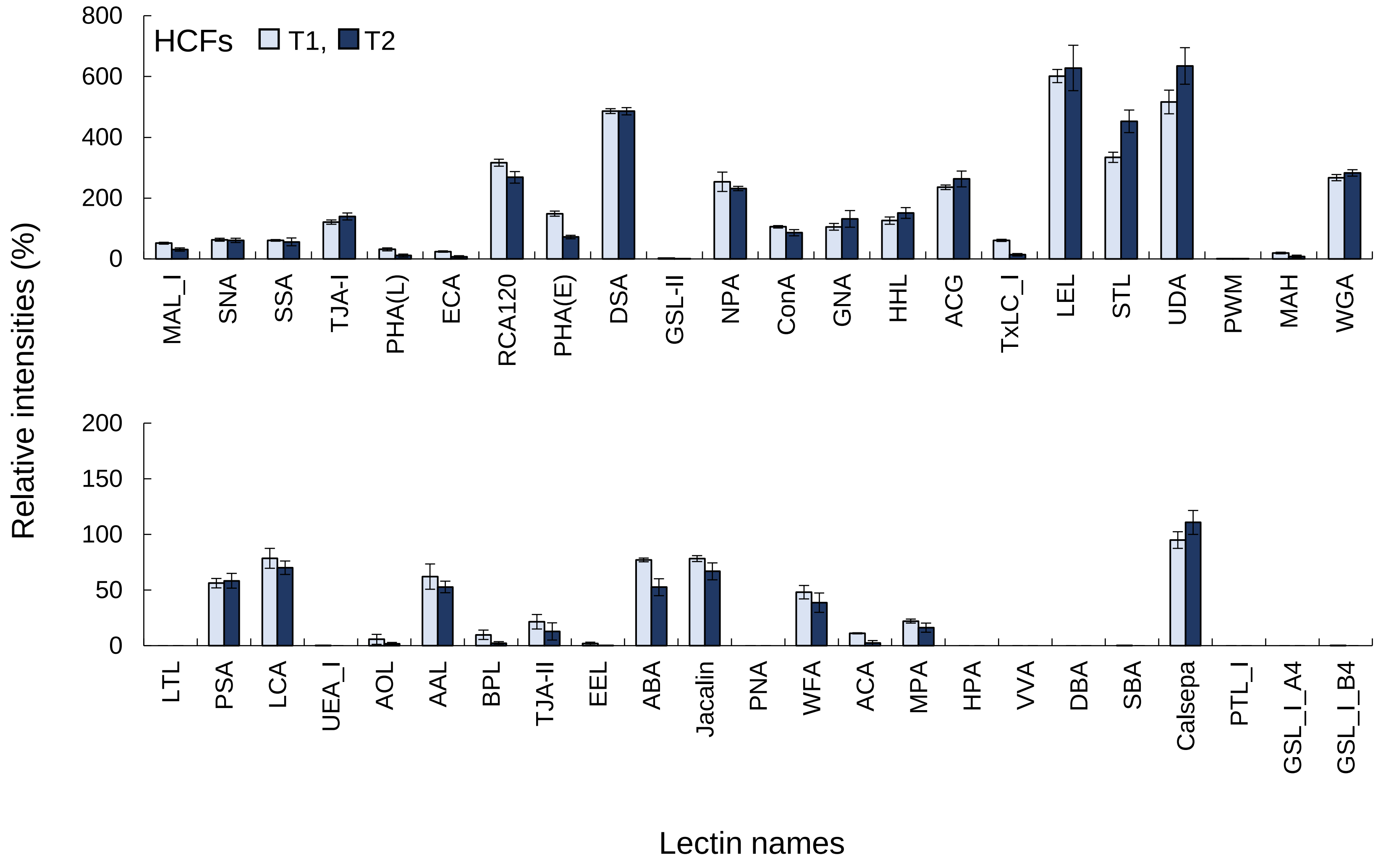

Supplemental Fig. S1 Itakura et al.

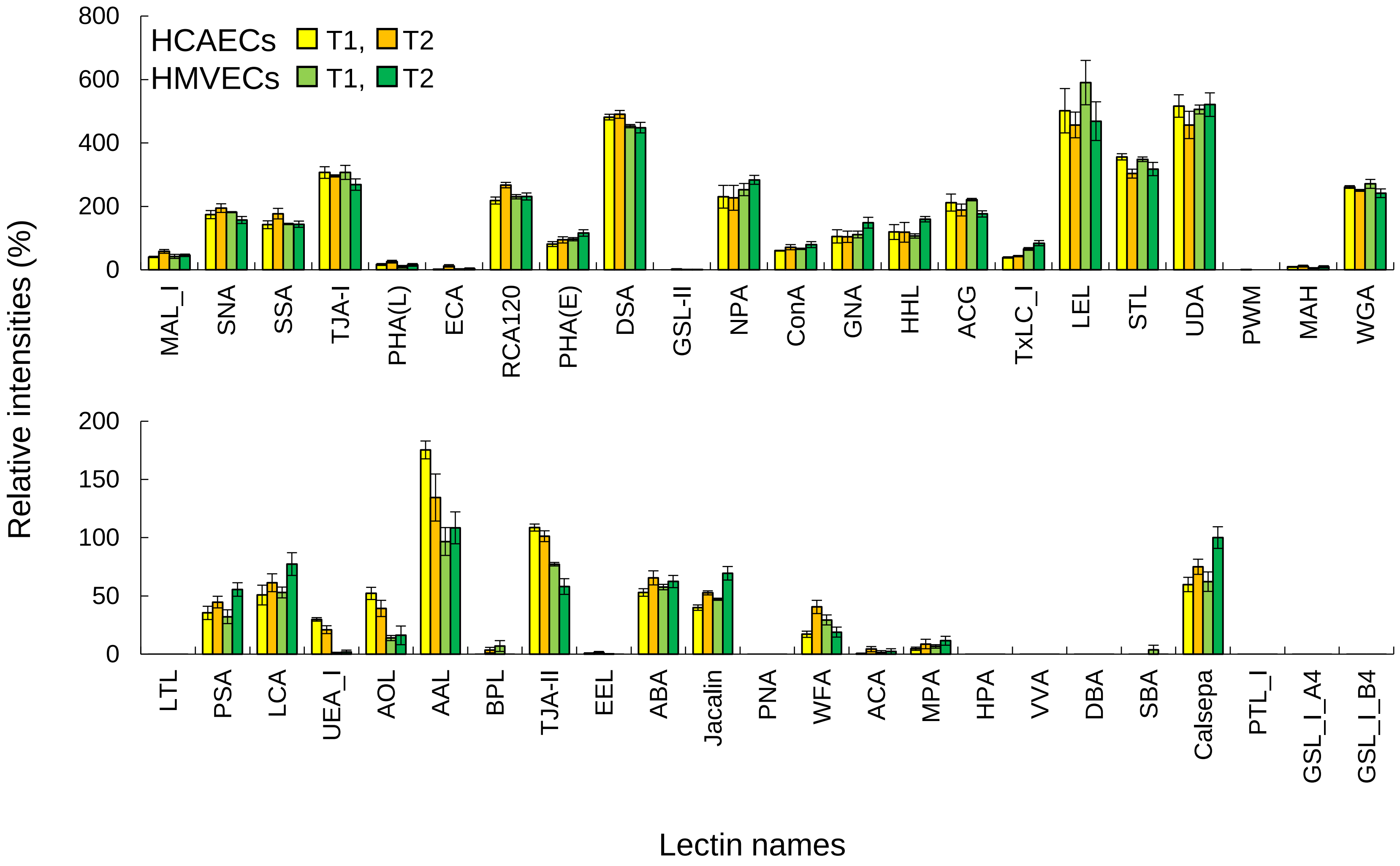

Supplemental Fig. S1 Itakura et al.

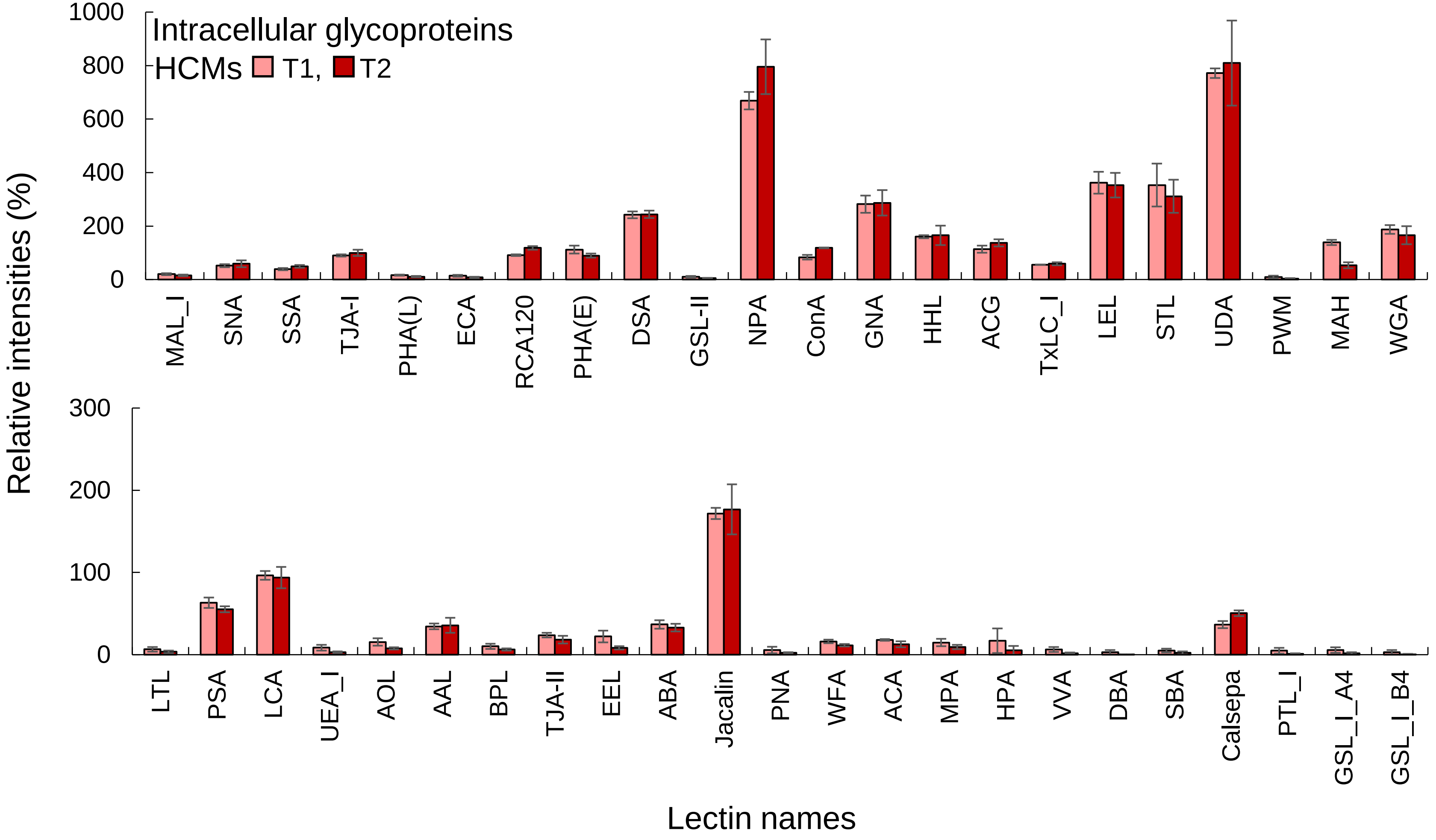

Supplemental Fig. S2 Itakura et al.

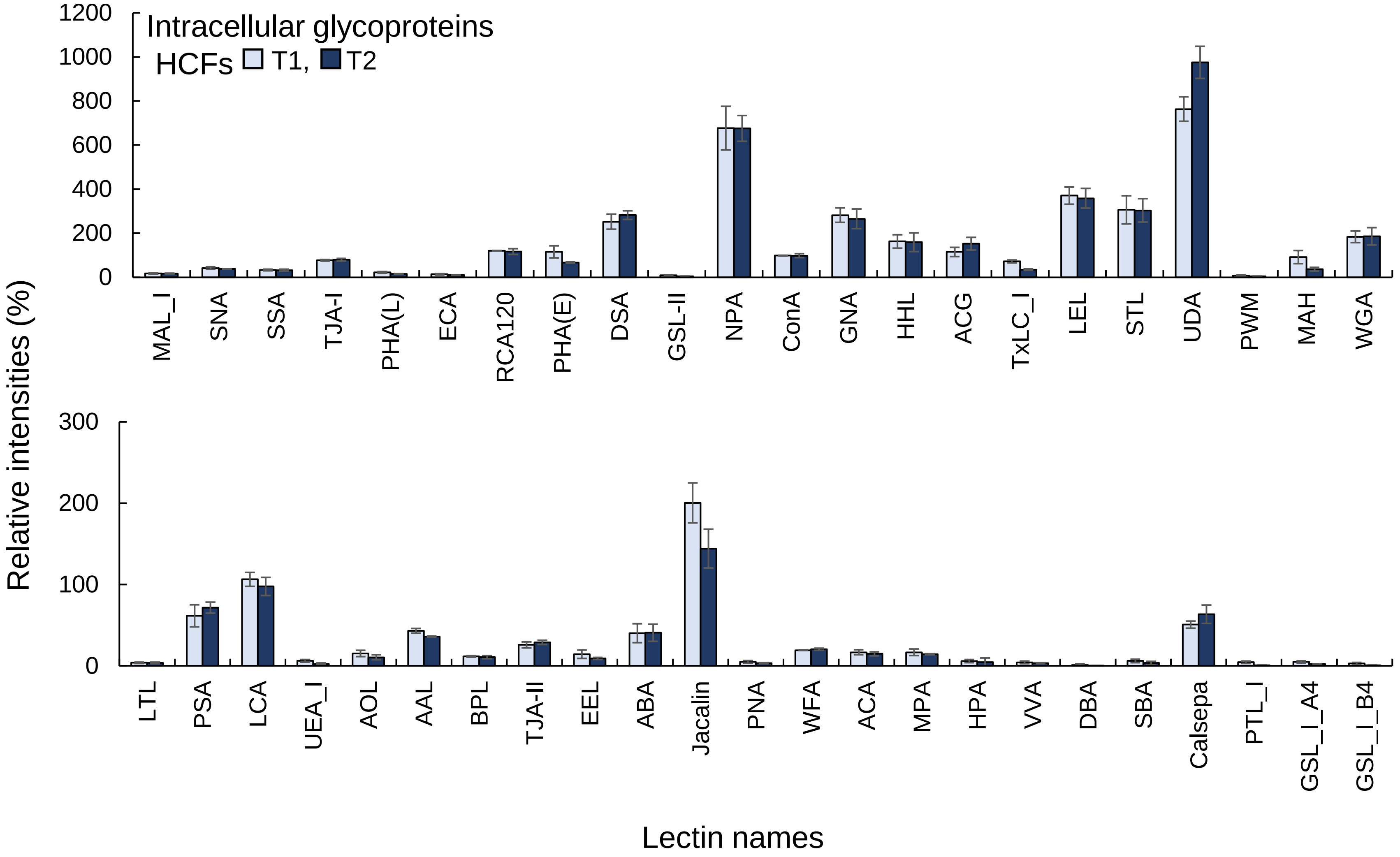

Supplemental Fig. S2 Itakura et al.

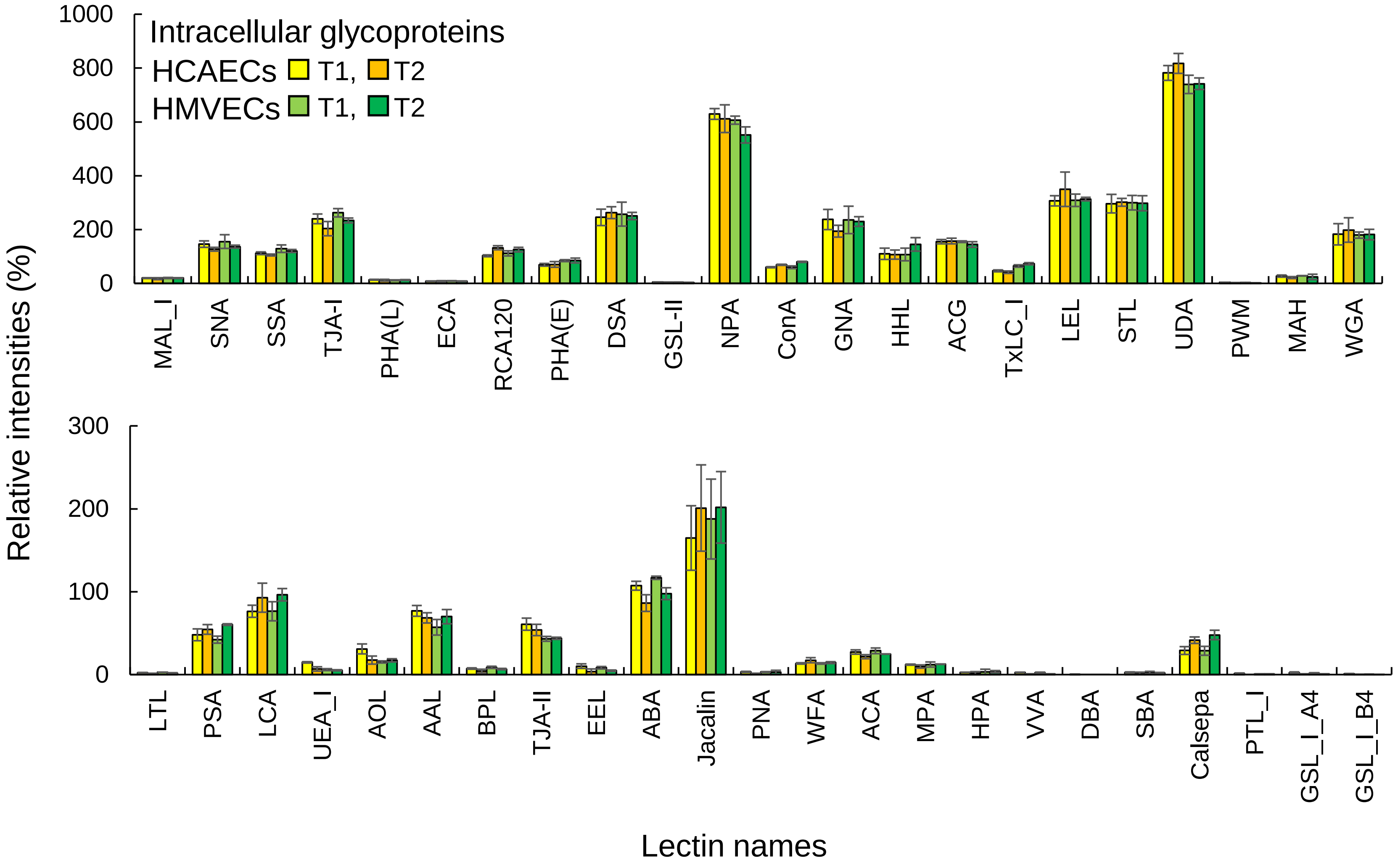

Supplemental Fig. S2 Itakura et al.

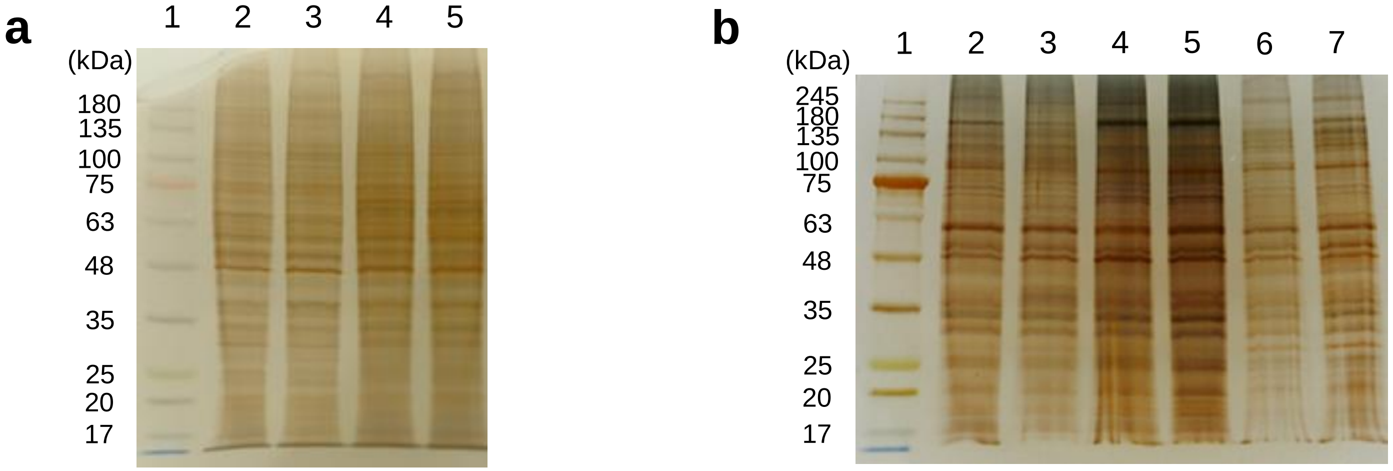

Supplemental Fig. S3 Itakura et al.

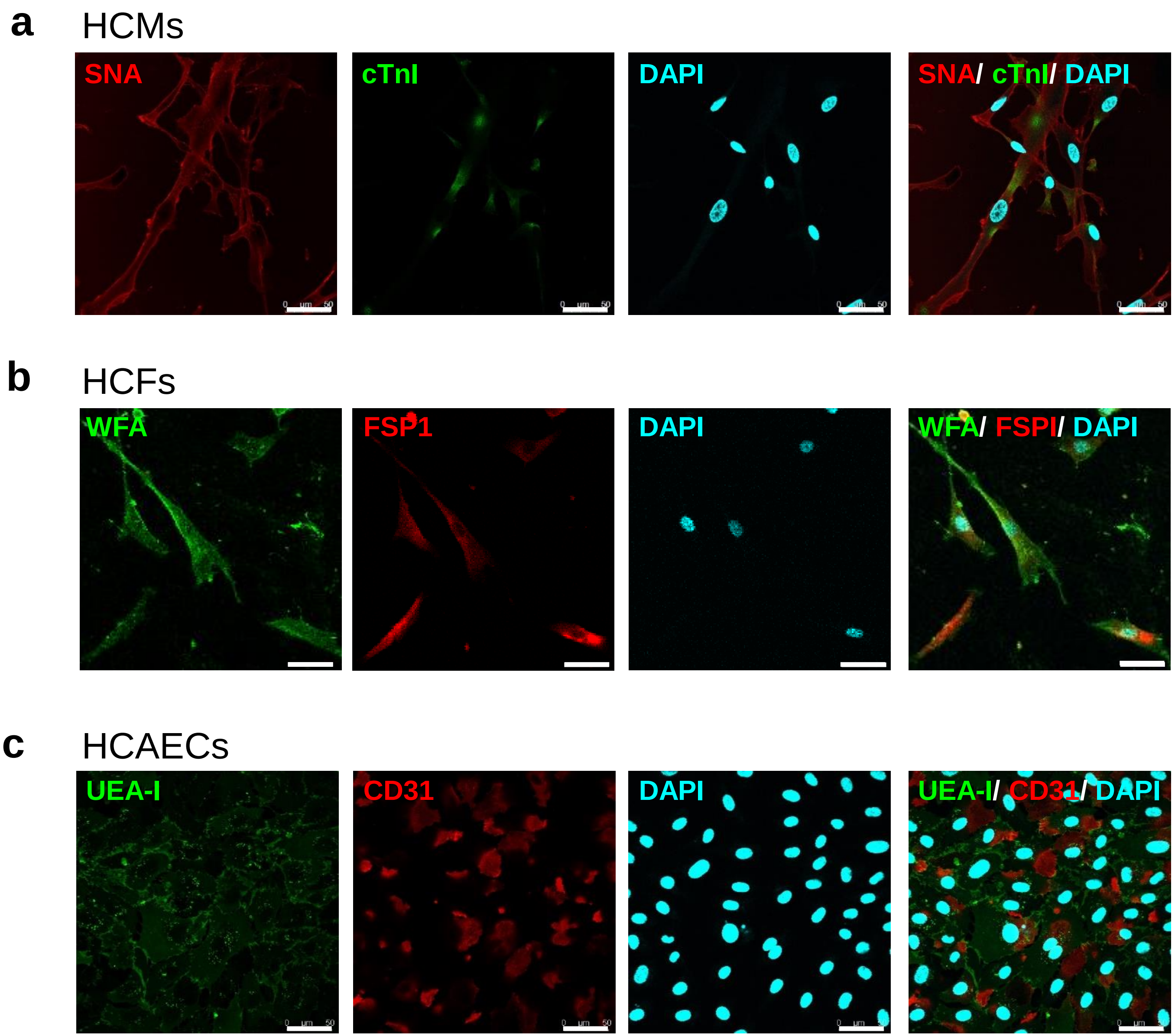

Supplemental Fig. S4 Itakura et al.

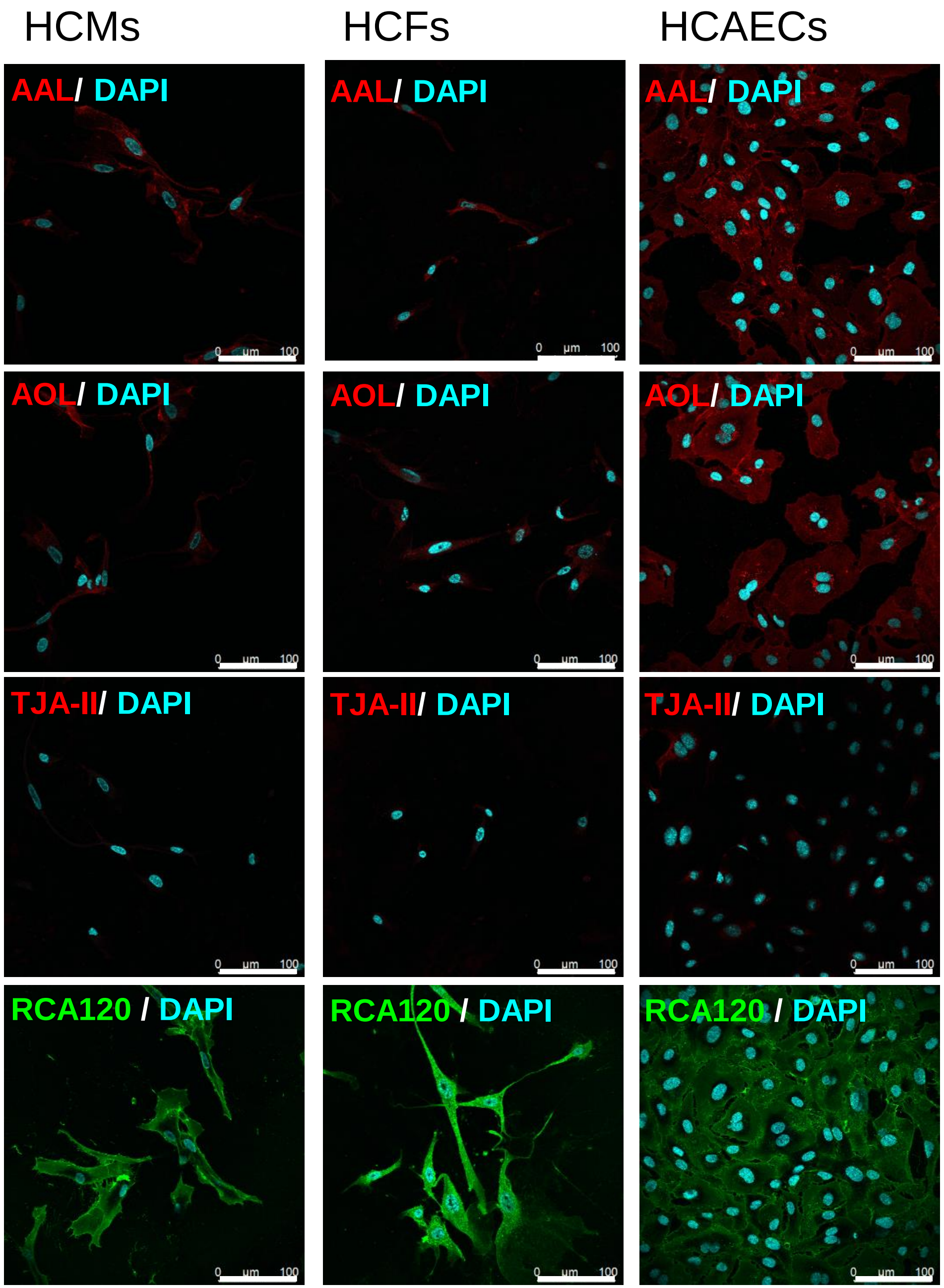

Supplemental Fig. S5 Itakura et al.

Supplemental Table S1. A List of lectins for the microarray.

Abbreviation	Lectin name	Glycan binding specificity[*1]	Reference No. of LfDB[*2]
LTL	*Lotus tetragonolobus*	Fucα1-3(Galβ1-4)GlcNAc, Fucα1-2Galβ1-4GlcNAc	LfDB0178
PSA	*Pisum sativum*	Fucα1-6GlcNAc, α-Man	LfDB0181
LCA	*Lens culinaris*	Fucα1-6GlcNAc, α-Man	LfDB0171
UEA-I	*Ulex europaeus*	Fucα1-2Galβ1-4GlcNAc	LfDB0185
AOL	*Aspergillus oryzae*	Fucα1-6GlcNAc, Fucα1-2Galβ1-4GlcNAc	LfDB0129
AAL	*Aleuria aurantia*	Fucα1-3(Galβ1-4)GlcNAc, Fucα1-6GlcNAc	LfDB0124
MAL-I	*Maackia amurensis*	Siaα2-3Galβ1-4GlcNAc	-
SNA	*Sambucus nigra*	Siaα2-6Gal/GalcNAc	LfDB0233
SSA	*Sambucus sieboldiana*	Siaα2-6Gal/GalcNAc	LfDB0232
TJA-I	*Trichosanthes japonica*	Siaα2-6Gal/GalcNAc	LfDB0224
PHA(L)	*Phaseolus vulgaris*	tri/tetra-antennary complex-type *N*-glycan	LfDB0180
ECA	*Erythrina cristagalli*	Galβ1-4GlcNAc	LfDB0175
RCA120	*Ricinus communis*	Galβ1-4GlcNAc	LfDB0231
PHA(E)	*Phaseolus vulgaris*	bi-antennary complex-type *N*-glycan, bisecting GlcNAc	LfDB0179
DSA	*Datura stramonium*	Galβ1-4GlcNAc, $(GlcNAc)_n$	LfDB0162
GSL-II	*Griffonia simplicifolia*	agalactosylated tri/tetra antennary glycans, GlcNAc	LfDB0176
NPA	*Narcissus pseudonarcissus*	Manα1-6Man	LfDB0208
ConA	*Canavalia ensiformis*	Manα1-6(Manα1-3)Man	-
GNA	*Galanthus nivalis*	Manα1-3Man	LfDB0206
HHL	*Hippeastrum hybrid*	Manα1-3Man, Manα1-6Man	LfDB0207
ACG	*Agrocybe cylindracea*	Galβ1-3Gal, Siaα2-3Galβ1-4Glc	LfDB0125
TxLCI	*Tulipa gesneriana*	Manα1-3(Manα1-6)Man, bi-antennary complex-type *N*-glycan	-
BPL	*Bauhinia purpurea alba*	Galβ1-3GalNAc	LfDB0173
TJA-II	*Trichosanthes japonica*	Fucα1-2Galβ	LfDB0225
EEL	*Euonymus europaeus*	Galα1-3Galβ1-4GlcNAc, Fucα1-2Galβ1-3GlcNAc	LfDB0222
ABA	*Agarics bisporus*	Galβ1-3GalNAc	LfDB0126
LEL	*Solanum lycopersicum*	$(GlcNAc\beta 1\text{-}4)_n$, $(Gal\beta 1\text{-}4GlcNAc)_n$	LfDB0164
STL	*Solanum tuberosum*	$(GlcNAc\beta 1\text{-}4)_n$	LfDB0167
UDA	*Urtica dioica*	$(GlcNAc\beta 1\text{-}4)_n$, High-Man	LfDB0168
PWM	*Phytolacca americana*	$(GlcNAc\beta 1\text{-}4)_n$	LfDB0165
Jacalin	*Artocarpus integrifolia*	Galβ1-3GalNAc, GalNAcα, GlcNAcβ1-3GalcNAc, Manα1-6(Manα1-3)Man	LfDB0146
PNA	*Arachis hypogaea*	Galβ1-3GalNAc	LfDB0172
WFA	*Wisteria floribunda*	GalNAcβ1-4GlcNAc, Galβ1-3GalNAc	LfDB0188
ACA	*Amaranthus caudatus*	Galβ1-3GalNAc, Siaα2-3Galβ1-3GalNAc	-
MPA	*Maclura pomifera*	GalNAcα, Galβ1-3GalNAc	-
HPA	*Helix pomatia*	GalNAcα	LfDB0105
VVA	*Vicia villosa*	GalNAcβ1-4Gal, GalNAcβ1-3Gal, GalNAcα	LfDB0187
DBA	*Dolichos biflorus*	GalNAcα1-3(Fuca1-2)Galβ, GalNAcα1-3GalNAc	LfDB0174
SBA	*Glycine max*	Tarminal GalNAc	LfDB0166
Calsepa	*Calystegia sepium*	Galactosylated bi-antennary complex-type *N*-glycan with bisecting GlcNAc, High-Man	LfDB0156
PTL-I	*Psophocarpus tetragonolobus*	GalNAcα, Galα1-3(Fucα1-2)Galβ	LfDB0182
MAH	*Maackia amurensis*	Siaα2-3Galβ1-3GalNAc	-
WGA	*Triticum vulgaris*	$(GlcNAc\beta 1\text{-}4)_n$, Hybrid type *N*-glycan with bisecting GlcNAc	LfDB0163
$GSL\text{-}I\text{-}A_4$	*Griffonia simplicifolia*	GalNAcα	LfDB0229
$GSL\text{-}I\text{-}B_4$	*Griffonia simplicifolia*	Galα	LfDB0230

Each lectin is showed as the abbreviation, the lectin name, and main specificity on LecChip (ver. 1.0).

[*1] This was modified from the Glyco Technica Ltd. web list. [*2] LfDB is shown from the web site (http://acgg.asia/lfdb2/index).

Supplemental Table S2. Lectin microarray data of membrane glycoproteins in heart constituent cells.

	HCMs									
Growth phase	T1					T2				
(N = 3)	1	2	3	Average	SD	1	2	3	Average	SD
LTL	0	0	0	0.0	0.0	0	0	0	0.0	0.0
PSA	67.6	45.2	74.4	62.4	15.3	52.7	45.9	46	48.2	3.9
LCA	90.5	55.6	102.5	82.9	24.4	85.4	68.6	66.4	73.5	10.4
UEA_I	0	0	0	0.0	0.0	0	0	0	0.0	0.0
AOL	3.9	0	0.4	1.4	2.1	3.4	0	0	1.1	2.0
AAL	85	86.5	46.3	72.6	22.8	57	54.9	0	37.3	32.3
MAL_I	52.7	28.7	56.4	45.9	15.0	39.5	48.5	50.6	46.2	5.9
SNA	132.9	109.1	155	132.3	23.0	140.9	136.6	28.4	102.0	63.7
SSA	116	76.6	133	108.5	28.9	117	110.5	28.1	85.2	49.6
TJA-I	231.5	233.4	248	237.6	9.0	221	210.9	128.1	186.7	51.0
PHA(L)	21.2	0	7.2	9.5	10.8	12	11.3	0	7.8	6.7
ECA	4.6	0	0	1.5	2.7	11.8	5.9	0	5.9	5.9
RCA120	239.9	197.8	222.8	220.2	21.2	276.9	270	220.8	255.9	30.6
PHA(E)	135.3	114.1	110	119.8	13.6	124.4	111.4	97.7	111.2	13.4
DSA	454.8	472.1	428.1	451.7	22.2	420.6	435.9	670.5	509.0	140.1
GSL-II	0	0	0	0.0	0.0	0	0	0	0.0	0.0
NPA	303	408.7	347.2	353.0	53.1	327.6	252.3	366.6	315.5	58.1
ConA	119.3	61.1	94.8	91.7	29.2	137.3	122.9	152.4	137.5	14.8
GNA	115	191.8	163.9	156.9	38.9	118.8	108.3	241.5	156.2	74.1
HHL	176.1	171.3	129.8	159.1	25.5	158.3	136.8	276.3	190.5	75.1
ACG	194.6	192.9	198	195.2	2.6	231.8	221.5	222.2	225.2	5.8
TxLC_I	78.7	71.6	70.5	73.6	4.5	90.3	69.1	14.9	58.1	38.9
BPL	0	0	0	0.0	0.0	0.3	0	0	0.1	0.2
TJA-II	20.7	0	4.3	8.3	10.9	16.9	9.7	0	8.9	8.5
EEL	0	0	0	0.0	0.0	0	0	0	0.0	0.0
ABA	81.4	57.5	67.6	68.8	12.0	60.9	49.1	32.1	47.4	14.5
LEL	536.1	518.7	400.3	485.0	73.9	553.4	626.2	560.3	580.0	40.2
STL	237.5	380.1	352.3	323.3	75.6	263.8	321.6	385.8	323.7	61.0
UDA	490.6	636.9	584.9	570.8	74.2	484.5	602.8	554.5	547.3	59.5
PWM	0	0	0	0.0	0.0	1.4	15.4	0.9	5.9	8.2
Jacalin	87.4	47.2	68.9	67.8	20.1	72.9	60.6	29.1	54.2	22.6
PNA	0	0	0	0.0	0.0	0	0	0	0.0	0.0
WFA	19.8	0	7.7	9.2	10.0	28.2	18.3	7	17.8	10.6
ACA	8.3	0	3.3	3.9	4.2	1	0	0	0.3	0.6
MPA	16	1	12.3	9.8	7.8	15.3	9.3	0	8.2	7.7
HPA	0	0	0	0.0	0.0	0	0	0	0.0	0.0
VVA	0	0	0	0.0	0.0	0	0	0	0.0	0.0
DBA	0	0	0	0.0	0.0	0	0	0	0.0	0.0
SBA	0	0	0	0.0	0.0	0	0	0	0.0	0.0
Calsepa	91.3	55.7	112.4	86.5	28.7	107.5	92.9	139	113.1	23.6
PTL_I	0	0	0	0.0	0.0	0	0	0	0.0	0.0
MAH	6.4	0	1.8	2.7	3.3	10.8	1.3	0	4.0	5.9
WGA	282	286.4	296	288.1	7.2	256.4	271.8	181	236.4	48.6
GSL_I_A4	0	0	0	0.0	0.0	0	0	0	0.0	0.0
GSL_I_B4	0	0	0	0.0	0.0	0.1	0	0	0.0	0.1

	HCFs									
Growth phase	T1					T2				
(N = 3)	1	2	3	Average	SD	1	2	3	Average	SD
LTL	0	0	0	0.0	0.0	0	0	0	0.0	0.0
PSA	52	64.5	52.2	56.2	7.2	51.4	52.1	71.6	58.4	11.5
LCA	60.7	86.2	88.8	78.6	15.5	65.3	63.1	82.1	70.2	10.4
UEA_I	0.9	0	0	0.3	0.5	0	0	0	0.0	0.0
AOL	14.6	0.1	2.5	5.7	7.8	4	1.2	0	1.7	2.1
AAL	82.8	60.2	43.4	62.1	19.8	63	49.4	46	52.8	9.0
MAL_I	56.5	52.7	45.5	51.6	5.6	40.7	26.5	23.7	30.3	9.1
SNA	52.6	68	68.2	62.9	8.9	74.3	56.8	50.3	60.5	12.4
SSA	66.6	57.7	58.9	61.1	4.8	71.4	64.9	31	55.8	21.7
TJA-I	134.3	109.7	118	120.7	12.5	138.3	119.8	160	139.4	20.1
PHA(L)	38.9	32	22.9	31.3	8.0	18.6	12.2	2.1	11.0	8.3
ECA	28	21.6	22.1	23.9	3.6	13.5	6.4	0	6.6	6.8
RCA120	339.3	300.2	310	316.5	20.3	283.7	290.4	231	268.4	32.5
PHA(E)	165	142.2	138	148.4	14.5	76.9	77.8	60.8	71.8	9.6
DSA	501.8	475.7	481.1	486.2	13.8	504.8	464.1	489.1	486.0	20.5
GSL-II	2	2.8	2.5	2.4	0.4	0	2.6	0	0.9	1.5
NPA	192.1	267.8	300.6	253.5	55.6	222.7	245.9	226.9	231.8	12.4
ConA	113.6	99.6	103.6	105.6	7.2	99.9	92	66.6	86.2	17.4
GNA	86.4	104.2	125.1	105.2	19.4	92.9	117.1	184.2	131.4	47.3
HHL	109.3	149.5	119.5	126.1	20.9	115.6	168.4	169.7	151.2	30.9
ACG	233.2	224.1	249.7	235.7	13.0	300	211.7	277.2	263.0	45.8
TxLC_I	68.3	58.3	56.5	61.0	6.4	16	19.4	5.4	13.6	7.3
BPL	18.3	5.4	5.6	9.8	7.4	4.9	1.7	0	2.2	2.5
TJA-II	34.7	14.7	15.2	21.5	11.4	26.9	11.7	0	12.9	13.5
EEL	4.4	0.8	0.4	1.9	2.2	0.4	0	0	0.1	0.2
ABA	77.7	73.9	79.6	77.1	2.9	67.2	49.4	41.4	52.7	13.2
LEL	643.3	591.4	570.2	601.6	37.6	649.2	745.3	489.4	628.0	129.3
STL	321.7	313.6	367.5	334.3	29.1	417.8	413.6	526.3	452.6	63.9
UDA	449.5	584.5	514.1	516.0	67.5	536.2	623.6	744.9	634.9	104.8
PWM	0.6	0	1.8	0.8	0.9	0.5	0	0	0.2	0.3
Jacalin	72.8	81.4	80.7	78.3	4.8	63.9	81.4	55.5	66.9	13.2
PNA	0	0	0	0.0	0.0	0	0	0	0.0	0.0
WFA	60.1	40.9	43.6	48.2	10.4	54.9	36.5	24.8	38.7	15.2
ACA	11.9	11	10.4	11.1	0.8	7	0.4	0	2.5	3.9
MPA	22.5	18.9	24.9	22.1	3.0	23.9	14.6	10.1	16.2	7.0
HPA	0	0	0	0.0	0.0	0	0	0	0.0	0.0
VVA	0	0	0	0.0	0.0	0	0	0	0.0	0.0
DBA	0	0	0	0.0	0.0	0	0	0	0.0	0.0
SBA	0	0	0.4	0.1	0.2	0	0	0	0.0	0.0
Calsepa	85.5	89.8	109.8	95.0	13.0	94.3	107.3	130.9	110.8	18.6
PTL_I	0	0	0	0.0	0.0	0	0	0	0.0	0.0
MAH	23.8	15	19.6	19.5	4.4	13.7	10.4	0	8.0	7.2
WGA	273.5	281.5	246.8	267.3	18.2	286.1	262.4	299	282.5	18.6
GSL_I_A4	0	0	0	0.0	0.0	0	0	0	0.0	0.0
GSL_I_B4	0.5	0	0	0.2	0.3	0	0	0	0.0	0.0

	HCAECs									
Growth phase	T1					T2				
(N = 3)	1	2	3	Average	SD	1	2	3	Average	SD
LTL	0	0	0	0.0	0.0	0	0	0	0.0	0.0
PSA	24.2	43.1	39.1	35.5	10.0	34.8	51.3	47.9	44.7	8.7
LCA	34.1	57.2	61.3	50.9	14.7	46	69.9	67.8	61.2	13.2
UEA_I	32.8	28.2	28.3	29.8	2.6	25.3	14.4	23.2	21.0	5.8
AOL	53	60.8	42.8	52.2	9.0	50.6	26.5	40.5	39.2	12.1
AAL	168.4	190.6	167.1	175.4	13.2	134.3	169.5	99.7	134.5	34.9
MAL_I	44.3	37.6	39.5	40.5	3.5	64.8	45.9	62.9	57.9	10.4
SNA	197.5	155.5	169.1	174.0	21.4	218.6	171.5	193.4	194.5	23.6
SSA	166.7	130.1	129.5	142.1	21.3	202	144.8	183.9	176.9	29.2
TJA-I	305.7	275.4	338.9	306.7	31.8	291.7	293.3	302.7	295.9	5.9
PHA(L)	21.7	16.2	12.4	16.8	4.7	33.2	18.8	25.2	25.7	7.2
ECA	3.6	2.1	0.4	2.0	1.6	13	5.8	18.4	12.4	6.3
RCA120	239.8	212	203.7	218.5	18.9	283.3	254	263.2	266.8	15.0
PHA(E)	85.2	91.8	66	81.0	13.4	112	78.5	92.8	94.4	16.8
DSA	464.8	484	494.9	481.2	15.2	482	514.2	473.9	490.0	21.3
GSL-II	0	0	0	0.0	0.0	0.2	0	5.1	1.8	2.9
NPA	158.7	271.1	261	230.3	62.2	157.6	293.4	230.1	227.0	68.0
ConA	61.7	57.6	61.4	60.2	2.3	80.7	54.5	78.3	71.2	14.5
GNA	63.7	123.8	128.5	105.3	36.1	69	116.2	126.5	103.9	30.7
HHL	75	153.4	128.8	119.1	40.1	77.1	178.9	98.7	118.2	53.6
ACG	262.9	173.4	199.3	211.9	46.1	226.2	165.3	173.7	188.4	33.0
TxLC_I	37.7	42.4	35.9	38.7	3.4	47.8	41.4	40.1	43.1	4.1
BPL	0	0	0	0.0	0.0	7.7	0	2.9	3.5	3.9
TJA-II	114.6	106.6	105	108.7	5.1	110.6	96.7	96.3	101.2	8.1
EEL	1.7	0	0	0.6	1.0	2.6	0	1.9	1.5	1.3
ABA	58.1	53.7	47.1	53.0	5.5	76.4	55.6	64.4	65.5	10.4
LEL	640.6	444.5	419.4	501.5	121.1	534.1	397.6	437.9	456.5	70.1
STL	355.1	339.7	373.4	356.1	16.9	277.9	305.1	327.2	303.4	24.7
UDA	446.4	560.3	542	516.2	61.2	384.4	533.3	451.6	456.4	74.6
PWM	0	0	0	0.0	0.0	0.4	0	0	0.1	0.2
Jacalin	35.5	43.2	41.1	39.9	4.0	54.5	49.2	54.3	52.7	3.0
PNA	0	0	0	0.0	0.0	0	0	0	0.0	0.0
WFA	22	16.6	12.9	17.2	4.6	51.1	31.7	39	40.6	9.8
ACA	1.6	0	0	0.5	0.9	6.6	0	6.4	4.3	3.8
MPA	6.7	5.4	1.9	4.7	2.5	14.7	1	10.4	8.7	7.0
HPA	0	0	0	0.0	0.0	0	0	0	0.0	0.0
VVA	0	0	0	0.0	0.0	0	0	0	0.0	0.0
DBA	0	0	0	0.0	0.0	0	0	0	0.0	0.0
SBA	0	0	0	0.0	0.0	0	0	0	0.0	0.0
Calsepa	49.3	59.4	70.5	59.7	10.6	68.9	68.2	87.9	75.0	11.2
PTL_I	0	0	0	0.0	0.0	0	0	0	0.0	0.0
MAH	6.7	10.2	10.4	9.1	2.1	11.8	6.5	16.1	11.5	4.8
WGA	260.1	254.2	268.6	261.0	7.2	248.1	247	255.7	250.3	4.7
GSL_I_A4	0	0	0	0.0	0.0	0	0	0	0.0	0.0
GSL_I_B4	0	0	0	0.0	0.0	0	0	0	0.0	0.0

	HMVECs									
Growth phase	T1					T2				
(N = 3)	1	2	3	Average	SD	1	2	3	Average	SD
LTL	0	0	0	0.0	0.0	0	0	0	0.0	0.0
PSA	20.8	41.1	34.6	32.2	10.4	46.2	66.2	54	55.5	10.1
LCA	43.7	57.7	57.5	53.0	8.0	57.7	87.1	87.2	77.3	17.0
UEA_I	0	2.4	0	0.8	1.4	1.2	4.8	0	2.0	2.5
AOL	12.7	10.8	18.2	13.9	3.8	30.7	15.1	2.8	16.2	14.0
AAL	99.3	74.7	116	96.7	20.8	118.7	81.3	125.2	108.4	23.7
MAL_I	29.2	47.8	49.8	42.3	11.4	42.3	53.6	42.2	46.0	6.6
SNA	178.4	182.9	184.2	181.8	3.0	136.3	175.2	159.6	157.0	19.6
SSA	140.7	145.1	147.4	144.4	3.4	152.5	154.3	123.8	143.5	17.1
TJA-I	267.4	309.3	344.3	307.0	38.5	249.1	253.4	303.6	268.7	30.3
PHA(L)	3.4	16.9	9.8	10.0	6.8	14.8	22.6	7.7	15.0	7.5
ECA	0	5.7	0	1.9	3.3	0	9.4	0	3.1	5.4
RCA120	221	243.9	226.7	230.5	11.9	244.6	239.9	209.7	231.4	18.9
PHA(E)	90.3	91.9	106.4	96.2	8.9	128.8	122.6	96.1	115.8	17.4
DSA	455.8	442.8	460.3	453.0	9.1	480.6	424.2	439.4	448.1	29.2
GSL-II	0	0.9	0	0.3	0.5	0	1.2	0	0.4	0.7
NPA	288.3	223.2	247.1	252.9	32.9	264.1	275.9	310.4	283.5	24.1
ConA	65.6	69.8	62.2	65.9	3.8	87.2	90.7	61	79.6	16.2
GNA	108.8	94.1	131	111.3	18.6	144.5	120.8	179.7	148.3	29.6
HHL	99.1	101.2	119.9	106.7	11.5	146.6	155.7	175.5	159.3	14.8
ACG	228.4	220.6	214.9	221.3	6.8	157	186.7	184.9	176.2	16.7
TxLC_I	73.1	65.3	59.1	65.8	7.0	98.4	81.9	71.6	84.0	13.5
BPL	2.1	16.4	2.5	7.0	8.1	0	0	0	0.0	0.0
TJA-II	74.3	78.1	79.2	77.2	2.6	70.5	55.9	47.5	58.0	11.6
EEL	0	0.2	0	0.1	0.1	0	0	0	0.0	0.0
ABA	61.7	57.3	53.6	57.5	4.1	58.8	72.5	55.8	62.4	8.9
LEL	725.8	550.8	494	590.2	120.8	572.7	470.9	362.4	468.7	105.2
STL	351.7	358.6	334.7	348.3	12.3	301.7	291.9	359.3	317.6	36.4
UDA	480.3	528.4	508	505.6	24.1	473	496.2	593	520.7	63.7
PWM	0	0	0	0.0	0.0	0	0	0	0.0	0.0
Jacalin	47.4	48.6	45.6	47.2	1.5	72.3	77.9	58.3	69.5	10.1
PNA	0	0	0	0.0	0.0	0	0	0	0.0	0.0
WFA	23.2	37.7	27.1	29.3	7.5	23.9	22.5	10.4	18.9	7.4
ACA	0	4.5	0	1.5	2.6	0	7	0	2.3	4.0
MPA	6	9.7	4.3	6.7	2.8	7.9	19	7.7	11.5	6.5
HPA	0	0	0	0.0	0.0	0	0	0	0.0	0.0
VVA	0	0	0	0.0	0.0	0	0	0	0.0	0.0
DBA	0	0	0	0.0	0.0	0	0	0	0.0	0.0
SBA	0	11.4	0	3.8	6.6	0	0	0	0.0	0.0
Calsepa	50.8	78.4	57.5	62.2	14.4	84.4	116.5	99.4	100.1	16.1
PTL_I	0	0	0	0.0	0.0	0	0	0	0.0	0.0
MAH	0.2	6.8	6.2	4.4	3.6	8.9	15.9	3.8	9.5	6.1
WGA	250.6	264.9	297.8	271.1	24.2	224.7	231.3	268	241.3	23.3
GSL_I_A4	0	0	0	0.0	0.0	0	0	0	0.0	0.0
GSL_I_B4	0	0	0	0.0	0.0	0	0	0	0.0	0.0

Each cell at the indicated growth phase was used for lectin microarray analysis.
The data were averaged at each phase (T1; growth phase, T2; growth arrest) after normalization (n =3).

Supplemental Table S3. Lectin microarray data of intracellular glycoproteins in heart constituent cells.

Growth phase	HCMs T1					HCMs T2				
(N = 3)	1	2	3	Average	SD	1	2	3	Average	SD
LTL	3.3	8.3	7.8	6.5	2.8	5	3.4	2.2	3.5	1.4
PSA	57.3	69.9	62.1	63.1	6.4	57.2	57.3	51	55.2	3.6
LCA	96.2	101.7	91.1	96.3	5.3	108	91	82.3	93.8	13.1
UEA_I	4.5	10	11	8.5	3.5	4	2.7	2	2.9	1.0
AOL	10.3	17.1	18.8	15.4	4.5	8.4	8.4	6.2	7.7	1.3
AAL	31.9	38.3	32.7	34.3	3.5	43.6	37.7	25.8	35.7	9.1
MAL_I	15.6	22	23	20.2	4.0	14.2	19.6	13.6	15.8	3.3
SNA	46.8	52	57.9	52.2	5.6	61.7	70.7	45.5	59.3	12.8
SSA	34.5	40.3	42.7	39.2	4.2	46.5	55.3	46.1	49.3	5.2
TJA-I	92.3	92.5	85.7	90.2	3.9	93.1	113	93.6	99.9	11.3
PHA(L)	14.6	17.1	18.6	16.8	2.0	10.6	14	8.3	11.0	2.9
ECA	9.9	15.4	17.2	14.2	3.8	10.2	8.9	5.6	8.2	2.4
RCA120	93.7	87.4	91.5	90.9	3.2	115.3	126.4	113.8	118.5	6.9
PHA(E)	114.5	95.9	125.8	112.1	15.1	98.1	84.5	86.5	89.7	7.3
DSA	245.8	252.9	227.7	242.1	13.0	248.3	228.7	255.2	244.1	13.7
GSL-II	7.6	9.7	13.3	10.2	2.9	5.3	6.8	4.1	5.4	1.4
NPA	659.6	641.3	705.2	668.7	32.9	764.7	910	712.4	795.7	102.4
ConA	82.8	74.8	92.3	83.3	8.8	116.3	118.6	120.1	118.3	1.9
GNA	303.8	297.6	244.5	282.0	32.6	246	338.8	275	286.6	47.5
HHL	163.2	153.7	164.1	160.3	5.8	131.1	203.9	161.7	165.6	36.6
ACG	113.5	100.6	127.2	113.8	13.3	126.4	151.8	132.3	136.8	13.3
TxLC_I	56.3	55.9	54.8	55.7	0.8	61	63.3	53	59.1	5.4
BPL	6.5	12.2	11.7	10.1	3.2	7.7	5.7	5.3	6.2	1.3
TJA-II	20.4	25.5	25.1	23.7	2.8	17.4	23.2	14.1	18.2	4.6
EEL	15.1	22.1	29.4	22.2	7.2	7.4	10.4	6.6	8.1	2.0
ABA	33.8	33.9	42.7	36.8	5.1	32.9	37.4	28.2	32.8	4.6
LEL	369	318.6	398.8	362.1	40.5	318.3	335.1	405.8	353.1	46.4
STL	433.4	354	272.5	353.3	80.5	339.7	240.5	353.6	311.3	61.7
UDA	788	775.4	752.3	771.9	18.1	814.2	648.2	966.5	809.6	159.2
PWM	4.7	10.8	13.8	9.8	4.6	4.8	3.9	2.5	3.7	1.2
Jacalin	177.9	172.8	164.4	171.7	6.8	208.8	148	173.5	176.8	30.5
PNA	1.1	8.4	7.7	5.7	4.0	2.9	2	1.6	2.2	0.7
WFA	13.5	17.1	17.7	16.1	2.3	11.8	12.5	9.7	11.3	1.5
ACA	17.1	19.2	17.7	18.0	1.1	16.3	12.8	8.9	12.7	3.7
MPA	9.7	16.1	18.3	14.7	4.5	12	9.6	6.7	9.4	2.7
HPA	9.4	34	7.4	16.9	14.8	11.3	3.8	1	5.4	5.3
VVA	3	7.9	8.1	6.3	2.9	1.7	2.5	0.8	1.7	0.9
DBA	0	4.4	4.8	3.1	2.7	0.5	0	0	0.2	0.3
SBA	2.4	6.3	6.5	5.1	2.3	3.7	2.6	0.3	2.2	1.7
Calsepa	31.6	38.9	39	36.5	4.2	54.5	47.9	49.2	50.5	3.5
PTL_I	0.9	7.1	6.4	4.8	3.4	1.6	0.9	0.1	0.9	0.8
MAH	127.9	144.1	145.4	139.1	9.8	64.4	54.5	41.5	53.5	11.5
WGA	175	206.2	181.8	187.7	16.4	189.8	180.9	127.7	166.1	33.6
GSL_I_A4	1.7	7	7.9	5.5	3.4	2.7	1.7	0.1	1.5	1.3
GSL_I_B4	0.2	3.7	5.4	3.1	2.7	0.5	0.8	0	0.4	0.4

Growth phase	HCFs T1					HCFs T2				
(N = 3)	1	2	3	Average	SD	1	2	3	Average	SD
LTL	4.3	4.4	3.1	3.9	0.7	4.8	3.4	3.1	3.8	0.9
PSA	55.2	77	52.4	61.5	13.5	68	79.4	67.5	71.6	6.7
LCA	101.8	116.4	100.9	106.4	8.7	102.7	105.4	84.9	97.7	11.1
UEA_I	5.9	7.9	5	6.3	1.5	2.8	3.4	1.3	2.5	1.1
AOL	11.5	19.1	15.6	15.4	3.8	7.1	11.7	13.1	10.6	3.1
AAL	41.4	46.5	41.5	43.1	2.9	36.1	35.5	36.5	36.0	0.5
MAL_I	15.2	19.7	16.7	17.2	2.3	14.3	18.2	17.5	16.7	2.1
SNA	36.3	46.4	42.9	41.9	5.1	37.5	39.3	37.7	38.2	1.0
SSA	34.3	35.3	29.5	33.0	3.1	28.1	36.9	31.2	32.1	4.5
TJA-I	73.7	80.1	78.1	77.3	3.3	83	72.1	82.7	79.3	6.2
PHA(L)	20.5	26.4	19.4	22.1	3.8	13.7	15.6	15.3	14.9	1.0
ECA	10.6	15.9	13.5	13.3	2.7	10.7	10.3	9	10.0	0.9
RCA120	121.8	120.5	119.9	120.7	1.0	107.4	131.6	111.5	116.8	13.0
PHA(E)	144.5	89.8	113.2	115.8	27.4	64.3	65.8	70.8	67.0	3.4
DSA	286.8	219.5	250.1	252.1	33.7	277.7	265.1	303.6	282.1	19.6
GSL-II	6.8	11.2	8	8.7	2.3	4.4	4.1	5.6	4.7	0.8
NPA	565.6	710.7	754.3	676.9	98.8	637.2	646.7	743.2	675.7	58.6
ConA	100	95.7	98.9	98.2	2.2	91.4	107.8	93.9	97.7	8.8
GNA	263.4	262.5	320	282.0	32.9	281.2	214.4	299.2	264.9	44.7
HHL	132.7	193.3	163	163.0	30.3	110.7	182.7	184.4	159.3	42.1
ACG	93.9	116.3	135.5	115.2	20.8	119	167.6	171.1	152.6	29.1
TxLC_I	73.9	77.3	64.3	71.8	6.7	35.2	37.4	31.5	34.7	3.0
BPL	11.5	13	11.2	11.9	1.0	9.1	10.7	12.8	10.9	1.9
TJA-II	21.9	29.4	26.3	25.9	3.8	26.2	31.3	29.5	29.0	2.6
EEL	9.9	20	13.5	14.5	5.1	7.8	9.7	10.4	9.3	1.3
ABA	27.1	45.2	48.7	40.3	11.6	28.9	44.3	49.2	40.8	10.6
LEL	354.2	342.6	415	370.6	38.9	316.4	352.3	405.4	358.0	44.8
STL	380.4	269	269.6	306.3	64.1	361.8	290.3	257.5	303.2	53.3
UDA	797.1	793.8	698.9	763.3	55.8	1025.5	1009.4	892	975.6	72.9
PWM	4.4	11	6.8	7.4	3.3	5.2	4.3	4.1	4.5	0.6
Jacalin	225.4	176.3	199.2	200.3	24.6	170.2	138.7	123.6	144.2	23.8
PNA	4.2	6.9	4	5.0	1.6	2.6	3.4	3.9	3.3	0.7
WFA	18.9	20	19.1	19.3	0.6	19.2	21.1	21.6	20.6	1.3
ACA	17.3	19.5	13.4	16.7	3.1	17.7	13.8	13.1	14.9	2.5
MPA	12.1	19.5	18.7	16.8	4.1	13.6	14.7	14.6	14.3	0.6
HPA	7.2	7.2	3.7	6.0	2.0	10.7	2.5	1.2	4.8	5.2
VVA	3.6	6.2	3.3	4.4	1.6	4.1	3.1	2.8	3.3	0.7
DBA	0.7	2.7	0	1.1	1.4	0.6	0	0.1	0.2	0.3
SBA	5.5	8.5	4.6	6.2	2.0	5.9	3.1	2.1	3.7	2.0
Calsepa	49.4	55.6	47.4	50.8	4.3	60	76.2	54.5	63.6	11.3
PTL_I	4.2	6.1	3.4	4.6	1.4	0.5	1.4	0.9	0.9	0.5
MAH	126.2	72.1	77	91.8	29.9	46.2	30.3	34	36.8	8.3
WGA	212.8	173	163.4	183.1	26.2	227.6	180.6	149.7	186.0	39.2
GSL_I_A4	3.9	6.5	4.4	4.9	1.4	2.6	2.8	2.3	2.6	0.3
GSL_I_B4	1.9	4.4	2.7	3.0	1.3	0.1	1.7	0.3	0.7	0.9

Growth phase	HCAECs T1					HCAECs T2				
(N = 3)	1	2	3	Average	SD	1	2	3	Average	SD
LTL	2.5	2.5	2	2.3	0.3	0	1.6	1.1	0.9	0.8
PSA	40.8	55.2	48.4	48.1	7.2	56.5	59.1	47.8	54.5	5.9
LCA	79.6	81.4	68.1	76.4	7.2	110.3	92.6	75.4	92.8	17.5
UEA_I	13.9	15.6	14.9	14.8	0.9	4	9	7.7	6.9	2.6
AOL	24.4	32.2	36.1	30.9	6.0	12.7	17.8	22.4	17.6	4.9
AAL	72.5	74	84.3	76.9	6.4	74.3	68.9	62.1	68.4	6.1
MAL_I	18.5	21.6	19.6	19.9	1.6	13.3	20.6	19.4	17.8	3.9
SNA	157.9	134	147.1	146.3	12.0	126.5	120.5	134.8	127.3	7.2
SSA	115.8	113.3	106.4	111.8	4.9	103.4	103.6	109.2	105.4	3.3
TJA-I	221	257.3	241.9	240.1	18.2	195.1	233.5	182.7	203.8	26.5
PHA(L)	12.2	15.1	13.9	13.7	1.5	8.4	14.9	12.7	12.0	3.3
ECA	7.8	8.3	9.3	8.5	0.8	4.8	9.4	9.1	7.8	2.6
RCA120	99	104.2	105.1	102.8	3.3	135.7	137.6	124.5	132.6	7.1
PHA(E)	71.8	72.2	63.9	69.3	4.7	82.3	63.1	65.9	70.4	10.4
DSA	226.5	229.9	280.2	245.5	30.1	247.5	287.8	253.4	262.9	21.8
GSL-II	5.1	5.5	5	5.2	0.3	0	3.2	4.5	2.6	2.3
NPA	620.3	615.3	652.8	629.5	20.4	570.9	595.2	670.1	612.1	51.7
ConA	58.6	60.9	62.6	60.7	2.0	68.1	67.8	71.6	69.2	2.1
GNA	195.2	252	265.5	237.6	37.3	171	214.1	197.6	194.2	21.7
HHL	87.3	128.3	114.8	110.1	20.9	96	126.5	99.3	107.3	16.7
ACG	159.2	145.5	160	154.9	8.2	158.7	146.7	167.3	157.6	10.3
TxLC_I	46.5	50.1	43.8	46.8	3.2	42.8	45.4	36.4	41.5	4.6
BPL	6.4	8	7.4	7.3	0.8	1.6	5.3	6	4.3	2.4
TJA-II	52.8	67.2	62.2	60.7	7.3	46.7	60.2	54.8	53.9	6.8
EEL	7.5	10	13.1	10.2	2.8	0	4.5	6.1	3.5	3.2
ABA	101.3	111.2	109.6	107.4	5.3	74.6	92.1	92.1	86.3	10.1
LEL	289.7	303	327.2	306.6	19.0	323.3	303.9	422.8	350.0	63.8
STL	334.7	286.8	267.7	296.4	34.5	296	318.4	290.2	301.5	14.9
UDA	807.3	786.4	753	782.2	27.4	858.8	789.2	802.9	817.0	36.9
PWM	3.4	3.9	4.7	4.0	0.7	0	1.6	2.9	1.5	1.5
Jacalin	207.9	154.8	132.1	164.9	38.9	258.4	187.3	157.3	201.0	51.9
PNA	2.8	3.9	2.8	3.2	0.6	0	1.2	1.2	0.8	0.7
WFA	12.7	13.5	14.2	13.5	0.8	13.9	19.9	18.2	17.3	3.1
ACA	29.7	27.8	24.5	27.3	2.6	23.6	22.6	18.9	21.7	2.5
MPA	11.6	11.5	12.7	11.9	0.7	7.6	11.4	10.3	9.8	2.0
HPA	2.5	2.7	3.1	2.8	0.3	0	3.7	1.2	1.6	1.9
VVA	2.3	2.7	2.8	2.6	0.3	0	0.8	0.7	0.5	0.4
DBA	0	0.2	0.6	0.3	0.3	0	0	0	0.0	0.0
SBA	3.2	2.5	2.2	2.6	0.5	0	2.5	2.2	1.6	1.4
Calsepa	26.3	34.6	26.4	29.1	4.8	40.1	45.9	38.5	41.5	3.9
PTL_I	1	2.1	0.8	1.3	0.7	0	0	0	0.0	0.0
MAH	30.1	29	23.2	27.4	3.7	22.6	24.8	17.9	21.8	3.5
WGA	227.9	160.6	159.3	182.6	39.2	250.6	164.8	180.2	198.5	45.7
GSL_I_A4	1.9	2.1	3.3	2.4	0.8	0	0.8	0.9	0.6	0.5
GSL_I_B4	0.5	0.9	1.4	0.9	0.5	0	0	0.1	0.0	0.1

Growth phase	HMVECs T1					HMVECs T2				
(N = 3)	1	2	3	Average	SD	1	2	3	Average	SD
LTL	2.5	2.9	3	2.8	0.3	1.9	1.3	2.2	1.8	0.5
PSA	43.8	37.4	45.3	42.2	4.2	59.8	59.8	61.6	60.4	1.0
LCA	89.8	69.8	69.8	76.5	11.5	88.1	102.9	98	96.3	7.5
UEA_I	6.7	6.5	4.8	6.0	1.0	5.1	5.8	4.8	5.2	0.5
AOL	15.1	14.3	16.8	15.4	1.3	15.6	19.4	16.6	17.2	2.0
AAL	68.1	50.6	52.7	57.1	9.6	79.9	65.3	64.8	70.0	8.6
MAL_I	19.3	19.5	22.5	20.4	1.8	18.5	20.7	21.3	20.2	1.5
SNA	184.4	138.9	143.2	155.5	25.1	130.3	137.7	140.9	136.3	5.4
SSA	144.5	117.4	124.7	128.9	14.0	126.6	115.6	119.2	120.5	5.6
TJA-I	245	271.8	271.7	262.8	15.4	224.3	236.1	241.8	234.1	8.9
PHA(L)	10.5	13.9	11.7	12.0	1.7	11.8	14.6	11.5	12.6	1.7
ECA	9.2	9.8	8.6	9.2	0.6	7	9.3	8.4	8.2	1.2
RCA120	107.1	122.5	105.8	111.8	9.3	118.4	134.6	124.2	125.7	8.2
PHA(E)	86.6	81.2	86.7	84.8	3.1	94	84.7	76.9	85.2	8.6
DSA	227.5	308.5	236	257.3	44.5	266.1	243.2	242.8	250.7	13.3
GSL-II	4	5.6	4.2	4.6	0.9	3.3	4.4	3.4	3.7	0.6
NPA	594.6	602.3	622.8	606.6	14.6	525.9	544.4	584.2	551.5	29.8
ConA	63.4	54.6	63.3	60.4	5.1	78.2	80.9	81.7	80.3	1.8
GNA	177.6	273.7	256.3	235.9	51.2	209.9	243.6	237.9	230.5	18.0
HHL	81.7	113.8	127.2	107.6	23.4	116.5	160.7	158.9	145.4	25.0
ACG	153	158.1	154.4	155.2	2.6	132.3	149.8	152	144.7	10.8
TxLC_I	69	65.5	59.9	64.8	4.6	77.2	73.4	69	73.2	4.1
BPL	8.6	7.5	10.1	8.7	1.3	7.4	6.8	5.8	6.7	0.8
TJA-II	39.8	44.6	45.3	43.2	3.0	44	45	42.6	43.9	1.2
EEL	6.7	9.2	9	8.3	1.4	4	5.6	5	4.9	0.8
ABA	117	114.7	118.5	116.7	1.9	89.4	101.5	102.2	97.7	7.2
LEL	324.9	283.1	319.1	309.0	22.6	318.3	306.2	315.6	313.4	6.4
STL	325.7	272.2	302.6	300.2	26.8	320.1	266.8	308.3	298.4	28.0
UDA	700.8	754.4	763.7	739.6	34.0	717.3	756.1	751.8	741.7	21.3
PWM	3.4	3.1	3.1	3.2	0.2	1.7	2	1.6	1.8	0.2
Jacalin	241.3	174.3	147.7	187.8	48.2	249.8	189.6	166	201.8	43.2
PNA	3.3	2.7	3.4	3.1	0.4	2.3	5.5	0.8	2.9	2.4
WFA	12.7	13.3	14.2	13.4	0.8	15.3	15.1	13.2	14.5	1.2
ACA	32.4	25.6	28.3	28.8	3.4	25.1	24.6	24.7	24.8	0.3
MPA	14.4	8.6	13.3	12.1	3.1	12.4	12.6	12.7	12.6	0.2
HPA	7	1.4	1.3	3.2	3.3	5	2.7	2.7	3.5	1.3
VVA	1.6	3	2.1	2.2	0.7	1	0	0.6	0.5	0.5
DBA	0	0	0	0.0	0.0	0	0	0	0.0	0.0
SBA	3.9	2.6	1.1	2.5	1.4	2.5	2.4	1.3	2.1	0.7
Calsepa	34.3	23.8	28.3	28.8	5.3	53.9	47.1	42.5	47.8	5.7
PTL_I	1.1	0.4	0.7	0.7	0.4	1.2	0.2	0	0.5	0.6
MAH	28.9	27.8	28.7	28.5	0.6	36.3	18	18.7	24.3	10.4
WGA	185.9	186.9	166.5	179.8	11.5	200.8	182.9	161.6	181.8	19.6
GSL_I_A4	2.3	1.6	1.4	1.8	0.5	0.9	0.9	0.2	0.7	0.4
GSL_I_B4	0.6	0.6	0	0.4	0.3	0.4	0.2	0	0.2	0.2

Each cell at the indicated growth phase was used for lectin microarray analysis.
The data were averaged at each phase (T1; growth phase, T2; growth arrest) after normalization (n =3).
