# Supplementary material for: Glycan characteristics of human heart constituent cells maintaining organ function: relatively stable glycan profiles in cellular senescence
Source: Biogerontology. 2021 Oct 12;22(6):623–37. doi: 10.1007/s10522-021-09940-z (PMC8566412; doi:10.1007/s10522-021-09940-z)
Supplement: Supplementary file 2 — Supplementary material 2 (PDF 88 kb) [file 10522_2021_9940_MOESM2_ESM.pdf]

Supplementary material

Journal name: *Biogerontology*

Title: Glycan characteristics of human heart constituent cells maintaining organ function: relatively stable glycan profiles in cellular senescence

Author names: Yoko Itakura, Norihiko Sasaki, and Masashi Toyoda*

*Correspondence: Masashi Toyoda,

Research Team for Geriatric Medicine (Vascular Medicine), Tokyo Metropolitan Institute of Gerontology, 35-2 Sakae-cho, Itabashi-ku, Tokyo 173-0015, Japan; Email: mtoyoda@tmig.or.jp; Tel.: +81-3-3964-3241

Supplementary figure legends

Supplemental Fig. S1 Membrane glycan profiles analyzed using lectin microarray in HCMs, HCFs, HCAECs, and HMVECs. Bar graph representing the signal intensities (%) for 45 lectins in HCMs (red), HCFs (blue), HCAECs (yellow), and HMVECs (green) at T1 (growth phase; light color) and T2 (growth arrest; dark color). The characteristics of the 45 lectins and the values of the signal intensities obtained in this analysis are shown in Supplemental Table S1 and S2, respectively. The data of T1 in Supplemental Fig. S1 are same as in Fig. 3a. The data are presented as the mean ± SD (n = 3). All experiments were performed in triplicate.

Supplemental Fig. S2 Intracellular glycan profiles analyzed using lectin microarray in HCMs, HCFs, HCAECs, and HMVECs. Bar graph representing the signal intensities (%) for 45 lectins in HCMs (red), HCFs (blue), HCAECs (yellow), and HMVECs (green) at T1 (growth phase; light color) and T2 (growth arrest; dark color). The characteristics of the 45 lectins and the values of the signal intensities obtained in this analysis are shown in Supplemental Tables S1 and S3, respectively. The data of T1 in Supplemental Fig. S2 are same as in Fig. 4. The data are presented as the mean ± SD (n = 3). All experiments were performed in triplicate.

Supplemental Fig. S3 Detection of protein extracts from heart constituent cells. (**a**) Whole cell extracts from HCMs, HCFs, HCAECs, and HMVECs at T1 (growth phase) were applied to lanes 2–5, respectively. The bands were detected with silver staining. A molecular marker was applied to lane 1. (**b**) The membrane extracts from HCMs, HCFs, and HCAECs at T1 (growth phase) and the corresponding extracts at T2 (growth arrest) were applied to lanes 2, 4, 6 and 3, 5, 7, respectively. The bands were detected with silver staining. A molecular marker was applied to lane 1. All experiments were performed with repetition.

Supplemental Fig. S4 Localization of characteristic glycans in heart constituent cells. Each cell type was stained with the characteristic lectins and cell characteristic markers. **a** HCMs (T1) stained with SNA (red), a cardiomyocyte marker (cTnI, green), and the overlay image. **b** HCFs (T1) stained with WFA (green), a fibroblast marker (FSP1, red), and the overlay image. **c** HCAECs (T1) stained with UEA-I (green), vascular endothelial cell marker (CD31, red), and the overlay image. Blue staining represents the nucleus. Scale bar = 50 μm. All experiments were performed with repetition.

Supplemental Fig. S5. Expression of characteristic glycans in heart constituent cells. HCMs (left), HCFs (middle), and HCAECs (right) at T1 stained with each of the characteristic lectins. Three cell types stained with AAL (red), AOL (red), TJA-II (red), or RCA120 (green) with nucleus (blue). Scale bar = 100 μm. All experiments were performed with repetition.
